# Solvent-Free Iron(III) Chloride-Catalyzed Direct Amidation of Esters

**DOI:** 10.3390/molecules25051040

**Published:** 2020-02-26

**Authors:** Blessing D. Mkhonazi, Malibongwe Shandu, Ronewa Tshinavhe, Sandile B. Simelane, Paseka T. Moshapo

**Affiliations:** 1 Research Centre in Synthesis and Catalysis, Department of Chemical Science, University of Johannesburg, P.O. Box 524, Auckland Park 2006, South Africa; 201585722@student.uj.ac.za (B.D.M.); 201305188@student.uj.ac.za (M.S.); 201424658@student.uj.ac.za (R.T.); 2 Department of Chemistry, University of Eswatini, Private Bag 4, Kwaluseni M201, Eswatini; bssimelane@uniswa.sz

**Keywords:** amidation, iron(III) chloride, amides, esters, solvent-free

## Abstract

Amide functional groups are prominent in a broad range of organic compounds with diverse beneficial applications. In this work, we report the synthesis of these functional groups via an iron(iii) chloride-catalyzed direct amidation of esters. The reactions are conducted under solvent-free conditions and found to be compatible with a range of amine and ester substrates generating the desired amides in short reaction times and good to excellent yields at a catalyst loading of 15 mol%.

## 1. Introduction

Amide functional groups are present in a plethora of natural and organic molecules with beneficial applications in the pharmaceutical, agrochemical, and textile industries. Examples include proteins; medicinal drugs such as apixaban, lenalidomide, and enzalutamide; as well as pesticides such as Boscalid^®^ [1,2,3,4,5]. In 2018, amide functional groups were present in 68% of new small molecule drugs approved by the FDA [6]. In general, amides are synthesized by a reaction between carboxylic acids and amines using activating reagents such as HATU, PPh_3_, and DCC in excess amounts [7]. Alternatively, carboxylic acids can be converted to their acid chloride or anhydride derivatives that readily react with amines to form the desired amide bonds. These reactions are often not catalytic and generate large quantities of undesired co-products. The synthesis of amides using greener and more atom economic reaction conditions is therefore an attractive approach to curb this problem [8,9]. Alternative amidation methodologies have been reported and some of these methods include the use of boron-based catalysts for efficient coupling of carboxylic acids at mild reaction temperatures; however, these reactions generate boronic acid side products [10,11,12]. Transition metal catalysts have also been used to couple amines and azides with a variety of substrates such as carboxylic acids, aldehydes, alcohols, alkynes and aryl halides [13,14,15]. Esters are key features in several organic synthesis substrates and have also been reported as useful substrates for amide coupling under acidic or basic conditions [16,17,18,19,20]. Some of the recent reports in direct ester amidation reactions include the use of lanthanum trifluoromethanesulfonate (Ln(Otf)_3_) in low catalyst loading as reported by Ohshima and co-workers [21]. Hu and co-workers reported the use of a nickel catalyst for reductive amidation of esters using nitroarenes [22]. Similar reductive coupling reactions using chromium catalysts have also been recently reported by Zeng and co-workers [23]. Nickel has also been employed by Newman and co-workers for direct amidation of methyl esters at high reaction temperatures [24]. Therefore, there is a growing interest in the use of esters as substrates for direct amidation reactions. This interest provides an opportunity for the investigation of more catalysts as well as different amino substrates that can be used to promote ester amidation [25,26]. In this study, we report the direct amidation of esters using relatively cheap and readily available FeCl_3_ catalyst [27,28,29] under solvent-free reaction conditions.

## 2. Results and Discussion 

**Scheme 1:**
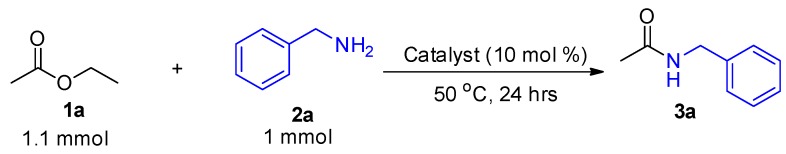
Catalyst = AlCl_3_, FeCl_3_, FeCl_2_, FeBr_3_ or BiCl_3_.

**Figure 1:**
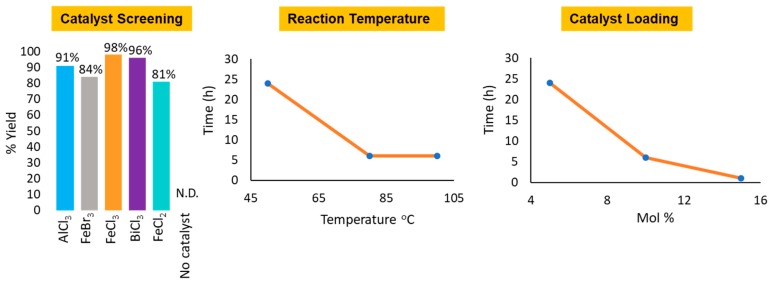
Reaction optimization. N.D. = Not Detected.

To establish the optimal reaction conditions for amidation of esters, readily available ethyl acetate and benzyl amine were selected as the ester and amine of choice, respectively (Scheme 1). Iron catalysts, namely, FeCl_3_, FeCl_2_, and FeBr_3_, were evaluated as suitable promoters for the direct amidation of 1a and 2a under solvent-free conditions at 50 °C. These catalysts were also compared to other readily available catalysts such as AlCl_3_ and BiCl_3_ and as can be seen in Figure 1, FeCl_3_ was found to be the most active catalyst as it afforded a higher yield of the desired product **3a**. A control experiment conducted at 50 °C in the absence of a catalyst did not form any product after 24 h. Thus, FeCl_3_ was selected as the preferred catalyst for further reaction optimization and evaluation. 

**Scheme 2:**
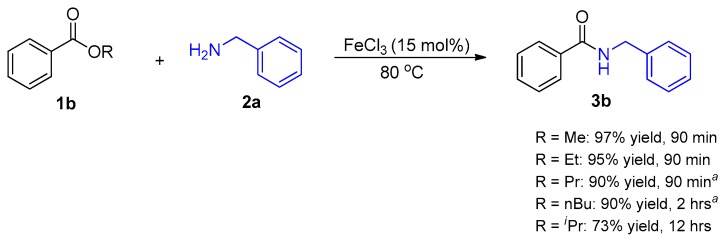
*^a^* Reaction mixture solidified and 0.5 mL CH_3_CN was added.

We then investigated the optimal reaction temperature and FeCl_3_ catalyst loading (Figure 1). Eighty degrees Celsius was the most favorable reaction temperature as the reaction went to completion within 5 h at 10 mol% catalyst loading. Increasing the reaction temperature to 100 °C did not effect a notable change in the rate of the reaction. Furthermore, increasing the catalyst loading to 15 mol% improved the rate of the reaction as TLC indicated completion of the reaction within 90 min. As a result, 15 mol% catalyst loading and 80 °C were selected as our optimal reaction conditions. In anticipation of reaction conditions that may require solvents (i.e., cases where both ester and amine substrates are solids or reactions that solidify upon mixing of substrates), we also investigated suitable solvents for FeCl_3_ catalyzed direct amidation of esters. We randomly selected four common organic solvents for investigation and the reactions between ester **1a** and amine **2a** (Scheme 1) were left to proceed for 90 min upon which TLC analysis indicated reaction completion for reactions conducted in acetonitrile and 1,2 dichloroethane (Table 1). Starting amine **2a** was observed in the tetrahydrofuran and toluene reactions after 90 min. Therefore, acetonitrile was found to be the most favorable solvent as it is relatively greener in comparison to 1,2 dichloroethane and it also afforded the product in high yield. The steric effect of alkoxy groups around the ester carbonyl carbon was also investigated (Scheme 2). An increase in steric bulk (isopropoxy) and chain length of the alkoxy group (n-butoxy) resulted in a decrease in ester reactivity and the reactions required longer reaction times to reach completion. However, there was no significant difference between esters with methoxy and ethoxy groups.

**Scheme 3:**
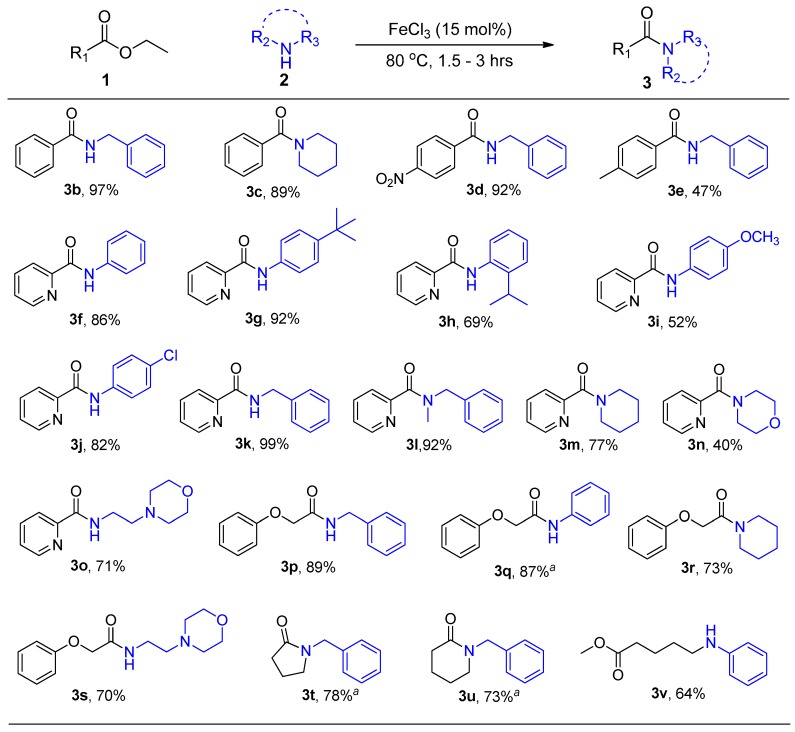
General reaction scheme for FeCl_3_ substrate scope evaluation. *^a^* Reaction mixture solidified and 0.5 mL of CH_3_CN was added.

Having established the optimal reaction conditions, we then investigated the catalyst’s reaction scope by varying both ester 1 and amine 2 substrates according to Scheme 3. Reactions were monitored by TLC. Aqueous work-up and flash column chromatography afforded the various amide products shown in Scheme 3. Aryl esters containing electron withdrawing groups were more active towards amidation than those with electron donating groups (product **3d** vs. **3e**). Both primary and secondary amines reacted to form amide products 3 in good yields. No reaction was observed between aryl ester **1b** (R = Et) and aniline. Interestingly, amidation of 2-pyridinecarboxylates with aryl amines was successful and afforded 2-pyridinecarboxamide products (**3f** to **3o**) in high yields. The formation of these pyridinecarboxamide products can be attributed to the possible coordination of the carbonyl oxygen and pyridine nitrogen to iron, thus, forming a stabilized intermediate complex that can undergo the observed amidation. 

To test the proposed nitrogen and oxygen iron coordination hypothesis, 3-pyridinecarboxylate was reacted with aniline and as expected, the reaction failed to afford the desired product. Reactions with 2-pyridinecarboxamide were successful with aryl amines possessing both electron donating and electron withdrawing groups as well as alkyl amines. No product formation was observed when 4-nitroaniline was used as a substrate and the starting materials were recovered. Phenyloxy esters were also found to be suitable substrates for direct amidation and afforded the desired amides in good yields ranging from 70% to 89% (**3p** to **3s**). 

**Scheme 4:**
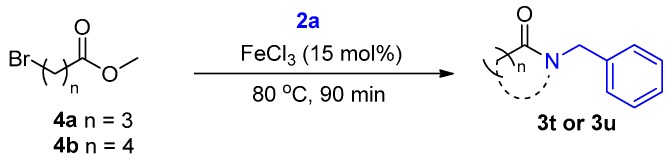
Lactam synthesis.

We further investigated the direct amidation of esters possessing alkyl bromide functional groups according to Scheme 4. Amidation of 4-bromobutanoate **4a** with benzyl amine **2a** afforded lactam **3t**. We then envisaged that increasing the alkyl chain length in Scheme 4 would enable us to synthesize lactams with varying ring sizes. 5-bromobutanoate **4b** afforded lactam **3u** in 73%; however, the reaction could not be replicated with 6-bromobutanoate as it formed several side products. Amidation using aniline afforded the substitution product **3v** which failed to undergo an intramolecular cyclization to form the desired lactam product. 

**Scheme 5:**
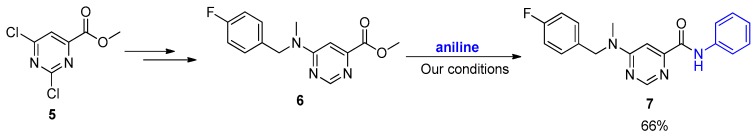
Application of the developed protocol in the synthesis of anti-TB pyrimidine carboxamides.

Finally, and having succeeded in the amidation of 2-pyridinecarboxylates, we then utilized the developed methodology in the synthesis of pyrimidine carboxamides **7**. Although their mode of action is unknown, these molecules are worth investigating due to their reported potent anti-tubercular activities against clinical *Mycobacterium tuberculosis* (*Mtb*) strains [30]. Direct amidation of the corresponding ester 6 synthesized from commercially available dichloropyrimidine **5** afforded the amide **7** in good isolated yields (Scheme 5). 

In conclusion, we have successfully demonstrated a solvent-free direct amidation of esters using FeCl_3_ as a Lewis acid catalyst at 80 °C. Amidation was effective with both primary and secondary amines and most of the reactions were complete in short reaction times (1.5–12 h) and good yields (47–99%). Linear and branched alkoxy groups on the corresponding esters could be displaced with ease to afford the desired amides. We have also demonstrated the synthesis of lactams with varying ring sizes from reactions between primary alkyl amines and 4-bromo or 5-bromoalkyl esters. The successful amidation of 2-pyridine carboxylates is currently being used in the synthesis of a library of novel aminopyrimidine carboxamides that will be screened for anti-TB activity.

## 3. Materials and Methods 

All the solvents used were freshly distilled and dried by appropriate techniques. All reagents were purchased from Sigma Aldrich FeCl_3_ reagent grade 97% was used as the catalyst. All reactions were monitored by thin layer chromatography (TLC) on aluminum-backed Merck silica gel 60 F254 plates using an ascending technique. The plates were visualized under UV light at 254 nm. Gravity column chromatography was done on Merck silica gel 60 (70–230 mesh). All proton nuclear magnetic resonance (^1^H NMR) spectra were recorded using deuterated chloroform solutions on a Bruker Ultrashield (400 or 500 MHz) spectrometer. Carbon-13 nuclear magnetic resonance (^13^C NMR) spectra were recorded on the same instruments at 100 MHz. All chemical shifts are reported in ppm. The chemical structures of the synthesized compounds were confirmed by comparison of their NMR data to literature reported data. Optimization reactions, ^1^H and ^13^C NMR data of all the compounds are available online as a Supplementary Materials.

*Typical Experimental Procedure for FeCl_3_-Catalyzed Direct Amidation of Esters*. An oven-dried pressure tube equipped with a magnetic stirrer was evacuated with nitrogen. To this was added of ester 1a (492 uL, 5.04 mmol), followed by amine 2a (0.5 mL, 4.58 mmol), and finally FeCl_3_ (111 mg, 0.684 mmol). The mixture was then sealed and stirred at 80 °C (0.5 mL of CH_3_CN was added if the reaction mixture solidified). The reaction was monitored by TLC until completion upon which it was diluted with EtOAc and washed once with saturated NaHCO_3_ and once with distilled H_2_O. The combined aqueous layers were extracted once with ethyl acetate. The combined organic layers were then dried over MgSO_4_, filtered and solvents removed under reduced pressure. The crude product was purified by silica gel flash column chromatography using a combination of hexane and ethyl acetate (3:2).

*N-Benzylacetamide (**3a**).* Yield: 99%; ^1^H NMR (400 MHz, CDCl_3_) δ 7.30–7.15 (m, 5H), 6.44 (br, 1H), 4.34 (d, *J* = 5.6 Hz, 2H), 1.94, (s, 3H); ^13^C NMR (100 MHz, CDCl_3_) δ 170.0, 138.2, 128.5, 127.6, 127.3, 43.5, 23.0. (The obtained NMR data agreed with the literature data for this compound.) [31] 

*N-Benzylbenzamide (**3b**).* Yield: 97%; ^1^H NMR (400 MHz, CDCl_3_) δ 7.36 (d, *J* = 7.2 Hz, 2H), 7.55–7.20 (m, 8H), 6.49 (s, 1H), 4.62 (d, *J* = 5.6 Hz, 2H); ^13^C NMR (100 MHz, CDCl_3_) δ 167.3, 138.2, 134.4, 131.5, 128.8, 128.6, 127.9, 127.6, 126.9, 44.1. (The obtained NMR data agreed with the literature data for this compound.) [32] 

*Phenyl(piperidin-1-yl)methanone (**3c**).* Yield: 89%; ^1^H NMR (400 MHz, CDCl_3_) δ7.36 (br, 5H), 3.68 (br, 2H), 3.30 (br, 2H), 1.70–1.40 (m, 6H). (The obtained NMR data agreed with the literature data for this compound.) [33] 

*N-(4-Nitrobenzyl)benzamide (**3d**).* Yield: 92%; ^1^H NMR (500 MHz, CDCl_3_) δ 8.20 (d, *J* = 8.5 Hz, 2H), 7.91 (d, *J* = 8.5 Hz, 2H), 7.40–7.23 (m, 5H), 6.83 (br, 1H), 4.60 (d, *J* = 5.5 Hz, 2H); ^13^C NMR (125 MHz, CDCl_3_) δ 165.4, 149.6, 139.9, 137.5, 128.9, 128.2, 127.9, 127.8, 123.7, 44.4. (The obtained NMR data agreed with the literature data for this compound.) [32] 

*N-(4-Methylbenzyl)benzamide (**3e**).* Yield: 47%; ^1^H NMR (500 MHz, CDCl_3_) δ 7.67 (d, *J* = 7.5 Hz, 2H), 7.40–7.18 (m, 7H), 6.51 (br, 1H), 4.60 (d, *J* = 5.0 Hz, 2H), 2.37 (s, 3H); ^13^C NMR (125 MHz, CDCl_3_) δ 167.3, 141.9, 138.4, 131.6, 129.2, 128.7, 127.9, 127.5, 127.0, 44.1, 21.4. (The obtained NMR data agreed with the literature data for this compound.) [32] 

*N-Phenylpicolinamide (**3f**).* Yield: 86%; ^1^H NMR (500 MHz, CDCl_3_) δ 10.1 (br, 1H), 8.59 (d, *J* = 3.2 Hz, 1H), 8.28 (d, *J* = 6.4 Hz, 1H), 7.92 – 7.83 (m, 1H), 7.77 (d, *J* = 6.0 Hz, 1H), 7.50 – 7.31 (m, 3H), 7.13 (t. *J* = 5.8 Hz, 1H); ^13^C NMR (100 MHz, CDCl_3_) δ 161.9, 149.8, 147.9, 137.7, 137.6, 129.0, 126.3, 124.2, 122.3, 119.6. (The obtained NMR data agreed with the literature data for this compound.) [34] 

*N-(4-(tert-Butyl)phenyl)picolinamide (**3g**).* Yield: 92%; ^1^H NMR (500 MHz, CDCl_3_) δ 9.97 (br, 1H), 8.57 (d, *J* = 4.0 Hz, 1H), 8.28 (d, *J* = 8.0 Hz, 1H), 7.84 (t, *J* = 7.8 Hz, 1H), 7.70 (d, *J* = 8.5 Hz, 2H), 7.48 – 7.36 (m, 3H), 1.32 (s, 9H); ^13^C NMR (125 MHz, CDCl_3_) δ 161.8, 149.9, 147.8, 147.1, 137.5, 135.1, 126.2, 125.9, 125.8, 122.2, 119.4, 34.6, 31.3. (The obtained NMR data agreed with the literature data for this compound.) [34] 

*(N-(2-Isopropylphenyl)picolinamide (**3h**).* Yield: 69%; ^1^H NMR (500 MHz, CDCl_3_) δ 10.2 (br, 1H), 8.62 (d, *J* = 4.0 Hz, 1H), 8.32 (d, *J* = 8.0 Hz, 1H), 8.22 (d, *J* = 8.0 Hz, 1H), 7.88 (t, *J* = 8.0 Hz, 1H), 7.51–7.12 (m, 4H), 3.34 – 3.20 (m, 1H), 1.34 (d, *J* = 7.0 Hz, 6H); ^13^C NMR (100 MHz, CDCl_3_) δ 162.1, 150.3, 148.1, 139,0, 137.5, 134.5, 126.5, 126.4, 125.6, 125.2, 122.4, 118.9, 28.2, 22.9. (The obtained NMR data agreed with the literature data for this compound.) [34] 

*N-(4-Methoxyphenyl)picolinamide (**3i**).* Yield: 52%; ^1^H NMR (500 MHz, CDCl_3_) δ 9.89 (br, 1H), 8.57 (d, *J* = 4.0 Hz, 1H), 7.86 (t, *J* = 8.0 Hz, 1H), 7.78 – 7.62 (m, 2H), 7.48–7.40 (m, 1H), 6.90 (d, *J* = 8.5 Hz, 2H), 3.78 (s, 3H); ^13^C NMR (125 MHz, CDCl_3_) δ 161.7, 156.4, 150.0, 147.9, 137.6, 131.0, 126.2, 122.2, 121.2, 114.2, 55.4. (The obtained NMR data agreed with the literature data for this compound.) [34] 

*N-(4-Chlorophenyl)picolinamide (**3j**).* Yield: 82%; ^1^H NMR (500 MHz, CDCl_3_) δ 9.98 (br, 1H), 8.52 (d, *J* = 3.5 Hz, 1H), 8.20 (d, *J* = 7.5 Hz, 1H), 7.82 (t, *J* = 7.5 Hz, 1H), 7.68 (d, *J* = 8.5 Hz, 2H), 7.40 (t, *J* = 5.6 Hz, 1H), 7.27 (d, *J* = 8.5 Hz, 2H); ^13^C NMR (100 MHz, CDCl_3_) δ 161.8, 149.4, 147.8, 137.5, 136.3, 129.0, 128.9, 126.4, 122.2, 120.8. (The obtained NMR data agreed with the literature data for this compound.) [34] 

*N-Benzylpicolinamide (**3k**).* Yield: 99%; ^1^H NMR (500 MHz, CDCl_3_) δ 8.52–8.38 (m, 2H), 8.20 (d, *J* = 8.0 Hz, 1H), 7.77 (t, *J* = 8.0 Hz, 1H), 7.45–7.20 (m, 6H), 4.63 (d, *J* = 6.0 Hz, 2H); ^13^C NMR (100 MHz, CDCl_3_) δ 164.0, 149.7, 147.8, 138.1, 137.1, 128.4, 127.6, 127.2, 125.9,122.1, 43.2. (The obtained NMR data agreed with the literature data for this compound.) [34] 

*N-Benzyl-N-methylpicolinamide (**3l**).* Yield: 92%; mixture of rotamers, ^1^H NMR (400 MHz, CDCl_3_) δ 8.52 (dd, *J* = 4.4 amd 14.0 Hz, 2H), 7.80 –7.58 (m, 4H), 7.35–7.10 (m, 12H), 4.72 (s, 2H), 4.62 (s. 2H), 2.97 (s, 3H), 2.91 (s, 3H); ^13^C NMR (100 MHz, CDCl_3_) δ 169.1, 168.8, 154.4, 154.3, 148.1, 136.8, 136.6, 128.5, 128.4, 128.0, 127.4, 127.3, 124.2, 123.5, 123.4, 54.4, 51.0, 36.3, 33.1. (The obtained NMR data agreed with the literature data for this compound.) [34] 

*Piperidin-1-yl(pyridin-2-yl)methanone (**3m**).* Yield: 77%; ^1^H NMR (500 MHz, CDCl_3_) δ 8.51 (d, *J* = 4.0 Hz, 1H), 7.70 (t, *J* = 7.8 Hz, 1H), 7.49 (d, *J* = 7.5 Hz, 1H), 7.30–7.15 (m, 1H), 3.81–3.65 (m, 2H), 3.31–3.48 (m, 2H), 1.72–1.43 (m, 6H); ^13^C NMR (100 MHz, CDCl_3_) δ 167.5, 154.7, 148.3, 136.8, 124.0, 123.1, 48.1, 43.2, 26.4, 25.4, 24.4. (The obtained NMR data agreed with the literature data for this compound.) [35] 

*Morpholino(pyridin-2-yl)methanone (**3n**).* Yield: 40%; ^1^H NMR (500 MHz, CDCl_3_) δ 8.50 (d, *J* = 4.0 Hz, 1H), 7.23 (t, *J* = 7.8 Hz, 1H), 7.60 (d, *J* = 7.5 Hz, 1H), 7.27 (t, *J* = 6.0 Hz, 1H), 3.88–3.50 (m, 8H); ^13^C NMR (125 MHz, CDCl_3_) δ 167.3, 153.6, 148.1, 137.0, 124.5, 124.0, 66.9, 66.6, 47.6, 42.7. (The obtained NMR data agreed with the literature data for this compound.) [36] 

*N-(2-morpholinoethyl)picolinamide (**3o**).* Yield: 71%; ^1^H NMR (500 MHz, CDCl_3_) δ 8.46 (d, *J* = 4.0 Hz, 1H), 8.25 (br, 1H), 8.08 (d, *J* = 8.0 Hz, 1H), 7.73 (t, *J* = 8.0 Hz, 1H), 7.31 (t, *J* = 6.0 Hz, 1H), 3.70–3.41 (m, 6H), 2.61–2.31 (m, 6H); ^13^C NMR (100 MHz, CDCl_3_) δ 164.2, 149.9, 147.9, 127.0, 125.8, 122.0, 66.8, 57.2, 53.3, 35.8. (The obtained NMR data agreed with the literature data for this compound.) [37] 

*N-Benzyl-2-phenoxyacetamide (**3p**).* Yield: 89%; ^1^H NMR (500 MHz, CDCl_3_) δ 7.48–7.21 (m, 7H), 7.10–6.87 (m, 4H); ^13^C NMR (100 MHz, CDCl_3_) δ 168.1, 157.1, 137.7, 129.7, 128.6, 127.6, 127.5, 122.1, 114.6, 67.3, 42.9. (The obtained NMR data agreed with the literature data for this compound.) [38] 

*2-Phenoxy-N-phenylacetamide (**3q**).* Yield: 87%; ^1^H NMR (500 MHz, CDCl_3_) δ 8.32 (bs, 1H), 7.59 (d, *J* = 8.0 Hz, 2H), 7.42–7.29 (m, 4H), 7.25–6.92 (m, 4H); ^13^C NMR (100 MHz, CDCl_3_) δ 166.2, 157.0, 136.8, 129.8, 129.0, 124.7, 122.3, 120.1, 114.8, 67.6. (The obtained NMR data agreed with the literature data for this compound.) [39] 

*2-Phenoxy-1-(piperidin-1-yl)ethanone (**3r**).* Yield: 70%; ^1^H NMR (500 MHz, CDCl_3_) δ 7.38–7.20 (m, 2H), 7.12–6.87 (m, 3H), 4.63 (s, 2H), 3.60–3.42 (m, 4H), 1.69–1.48 (m, 6H); ^13^C NMR (100 MHz, CDCl_3_) δ 166.0, 157.9, 129.4, 121.3, 114.5, 67.6, 46.3, 43.0, 26.3, 25.4, 24.3.

*N-(2-Morpholinoethyl)-2-phenoxyacetamide (**3s**).* Yield: 70%; ^1^H NMR (500 MHz, CDCl_3_) δ 7.37–7.25 (m, 2H), 7.09 (br, 1H), 7.09–6.70 (m, 3H), 4.45 (s, 2H), 3.65–3.47 (m, 4H), 3.44–3.35 (m, 2H), 2.52–2.27 (m, 6H); ^13^C NMR (100 MHz, CDCl_3_) δ 168.0, 157.2, 129.6, 121.9, 67.2, 66.8, 56.5, 53.0, 35.1. 

*1-Benzylpyrrolidin-2-one (**3t**).* Yield: 78%; ^1^H NMR (400 MHz, CDCl_3_) δ 7.24–7.71 (m, 5H), 4.35 (s, 2H), 3.15 (t, *J* = 6.8 Hz, 3H), 2.33 (t, *J* = 8.0 Hz, 2H), 1.91–1.83 (m, 2H); ^13^C NMR (100 MHz, CDCl_3_) δ 174.1, 135.9, 127.9, 127.3, 126.7, 45.8, 45.7, 30.1, 16.9. (The obtained NMR data agreed with the literature data for this compound.) [39] 

*1-Benzylpiperidin-2-one (**3u**).* Yield: 73%; ^1^H NMR (500 MHz, CDCl_3_) δ 7.35–7.18 (m, 5H), 4.53 (s, 2H), 3.13 (t, *J* = 5.0 Hz, 2H), 2.59–2.31 (m, 2H), 1.81–1.67 (m, 4H); ^13^C NMR (125 MHz, CDCl_3_) δ 169.7, 137.11, 128.4, 127.8, 127.1, 49.9, 47.1, 32.2, 23.0, 21.2. (The obtained NMR data agreed with the literature data for this compound.) [39] 

*Methyl 5-(phenylamino)pentanoate (**3v**).* Yield: 64%; ^1^H NMR (500 MHz, CDCl_3_) δ 7.16 (t, *J* = 7.8 Hz, 2H), 6.68 (t, *J* = 7.0 Hz, 1H), 6.59 (d, *J* = 7.5 Hz, 2H), 3.66 (s, 3H), 3.27 (t, *J* = 6.8 Hz, 1H), 3.12 (t, *J* = 7.0 Hz, 1H), 2.45–2.30 (m, 2H), 1.82–1.59 (m, 4H); ^13^C NMR (100 MHz, CDCl_3_) δ 173.7, 148.1, 129.2, 129.1, 117.2, 112.7, 51.4, 43.4, 33.7, 33.6, 28.8, 26.5, 22.4. (The obtained NMR data agreed with the literature data for this compound.) [40] 

*6-((4-Fluorobenzyl)(methyl)amino)-N-phenylpyrimidine-4-carboxamide* (**7**). Yield: 66%; Clear oil; ^1^H NMR (400 MHz, CDCl_3_) δ 9.94 (s, 1H), 8.62 (s, 1H), 7.74 (d, *J* = 8.0 Hz, 2H), 7.31–7.43 (m, 3H), 7.10–7.28 (m, 3H), 6.90–7.08 (m, 2H), 4.86 (br, 2H), 3.11 (br, 3H). ^13^C NMR (100 MHz, CDCl_3_) δ 163.2, 161.4, 157.1, 155.2, 137.4, 129.1, 124.6, 119.7, 115.8, 115.6, 100.3, 33.40. ^19^F NMR (376 MHz, CDCl_3_) δ -114.8 (The obtained NMR data agreed with the literature data for this compound.) [30] 

## Figures and Tables

**Table 1:** Solvent evaluation.

Entry	Solvent	Product Yield
1	Toluene	84% *^a^*
2	Acetonitrile	96%
3	Tetrahydrofuran	85% *^a^*
4	1,2 Dichloroethane	94%
5	No Solvent	99%

Reaction conditions: ester **1a** (1.1 mmol), amine **2a** (1 mmol), 15 mol% FeCl_3_, 80 ^o^C. *^a^* Reaction did not reach completion within 90 min.

## Supplementary Materials

The following are available online. Materials and methods, results of optimization conditions as well as NMR spectra of products.
