# Supplementary material for: Solvent-Free Iron(III) Chloride-Catalyzed Direct Amidation of Esters
Source: Molecules. 2020 Feb 26;25(5):1040. doi: 10.3390/molecules25051040 (PMC7179140; doi:10.3390/molecules25051040)
Supplement: Supplementary file 1 [file molecules-25-01040-s001.pdf]

Supporting Information

Solvent-free iron(III) chloride catalyzed direct amidation of esters

Blessing D Mkhonazi [1], Malibongwe Shandu[1], Ronewa Tshinavhe[1], Sandile B Simelane [2] and Paseka T Moshapo[1,*]

[1] Research Centre in Synthesis and Catalysis, Department of Chemical Science, University of Johannesburg, PO Box 524, Auckland Park, 2006, South Africa; 201585722@student.uj.ac.za (B.D.M); 201305188@student.uj.ac.za (M.S); 201424658@student.uj.ac.za (R.T).

[2] Department of Chemistry, University of Eswatini, Private Bag 4, Kwaluseni, M201,, Eswatini; bssimelane@uniswa.sz

*Correspondence: pasekam@uj.ac.za; Tel.: +27-11-559-2372 (P.T.M)

Table of Contents

1. Materials and Methods S2

2. Optimization of reaction conditions S2

3. Typical experimental procedure for $FeCl_3$ catalyzed direct amidation of esters S4

4. NMR Spectra of Products S5

1. Materials and Methods

All the solvents used were freshly distilled and dried by appropriate techniques. All reagents were purchased from Sigma Aldrich $FeCl_3$ reagent grade 97% was used as the catalyst. All reactions were monitored by thin layer chromatography (TLC) on aluminum-backed Merck silica gel 60 F254 plates using an ascending technique. The plates were visualized under UV light at 254 nm. Gravity column chromatography was done on Merck silica gel 60 (70–230 mesh). All proton nuclear magnetic resonance (^1H NMR) spectra were recorded using deuterated chloroform solutions on a Bruker Ultrashield (400 or 500 MHz) spectrometer. Carbon-13 nuclear magnetic resonance (^{13}C NMR) spectra were recorded on the same instruments at 100 MHz. All chemical shifts are reported in ppm. The chemical structures of the synthesized compounds were confirmed by comparison of their NMR data to literature reported data.

2. Optimization of reaction conditions

2.1 Catalyst Screening

Catalyst screening, reaction temperature as well as catalyst loading were optimized using ester **1a** and amine **2a** as model substrates. Reactions were carried out as reported in the manuscript using 10 mol% of each catalyst under solvent-free conditions. The reactions were left to stir for 24 hours at 50 °C. The formed products were isolated by column chromatographic techniques and the yields reported as shown in table S1. No product was observed upon TLC analysis of the control experiment.

Table S1

Entry	Catalyst	Temperature (°C)	Time (h)	Yield
1	$AlCl_3$	50	24	91%
2	$FeBr_3$	50	24	84%
3	$FeCl_3$	50	24	98%
4	$BiCl_3$	50	24	96%
5	$FeCl_2$	50	24	81%
6	None	50	24	0%

2.2 Reaction Temperature

Amidation of ester **1a** with amine **2a** was conducted at 50 °C, 80 °C and 100 °C as shown in Table S2. The reaction time to reach completion was monitored by TLC. Lowering the reaction temperature resulted in prolonged reaction times and there was no difference between reactions at 80 and 100 °C.

Table S2

Entry	Temperature (^{o}C)	Catalyst Loading (mol%)	Reaction Time (h)
1	100	10	6
2	80	10	6
3	50	10	24

2.3 Catalyst Loading

Amidation of ester **1a** with amine **2a** was conducted according to the procedure reported in the manuscript using 5 mol%, 10 mol% and 15 mol% $FeCl_3$. The reaction progress was monitored by TLC plate and the reaction times are shown in Tables S3. The optimum catalyst loading was 15 mol% as the reaction was complete within 1.5 hours.

Table S3

Entry	Catalyst Loading (mol%)	Temperature (^{o}C)	Reaction Time (h)
1	5	80	24
2	10	80	6
3	15	80	1.5

2.4 Solvent Screening

An oven-dried pressure tube equipped with a magnetic stirrer was evacuated with nitrogen. To this was added of ester **1a** (492 ul, 5.04 mmol) followed by amine **2a** (0.5 ml, 4.58 mmol) and a 1 ml of solvent (Toluene, acetonitrile, tetrahydrofuran or 1,2-dichloroethane. Finally, $FeCl_3$ (111 mg, 0.684 mmol) was added and the reaction mixture was then sealed and stirred at 80 ^{o}C. The reaction was left to stir for 90 min and was diluted with EtOAc and washed once with saturated $NaHCO_3$ and once with distilled H_2O. The combined aqueous layers were extracted once with ethyl acetate. The combined organic layers were then dried over $MgSO_4$, filtered and solvents removed under reduced pressure. The crude product was purified by silica gel flash column chromatography using a combination of hexane and ethyl acetate (3:2). The isolated product yields are reported in Table 1 of the manuscript.

3. Typical experimental procedure for $FeCl_3$ catalyzed direct amidation of esters

An oven-dried pressure tube equipped with a magnetic stirrer was evacuated with nitrogen. To this was added of ester **1a** (492 ul, 5.04 mmol) followed by amine **2a** (0.5 ml, 4.58 mmol) and finally $FeCl_3$ (111 mg, 0.684 mmol). The mixture was then sealed and stirred at 80 °C (0.5 ml of CH_3CN was added if the reaction mixture solidified). The reaction was monitored by TLC until completion upon which it was diluted with EtOAc and washed once with saturated $NaHCO_3$ and once with distilled H_2O. The combined aqueous layers were extracted once with ethyl acetate. The combined organic layers were then dried over $MgSO_4$, filtered and solvents removed under reduced pressure. The crude product was purified by silica gel flash column chromatography using a combination of hexane and ethyl acetate (3:2).

4. NMR Spectra of Products

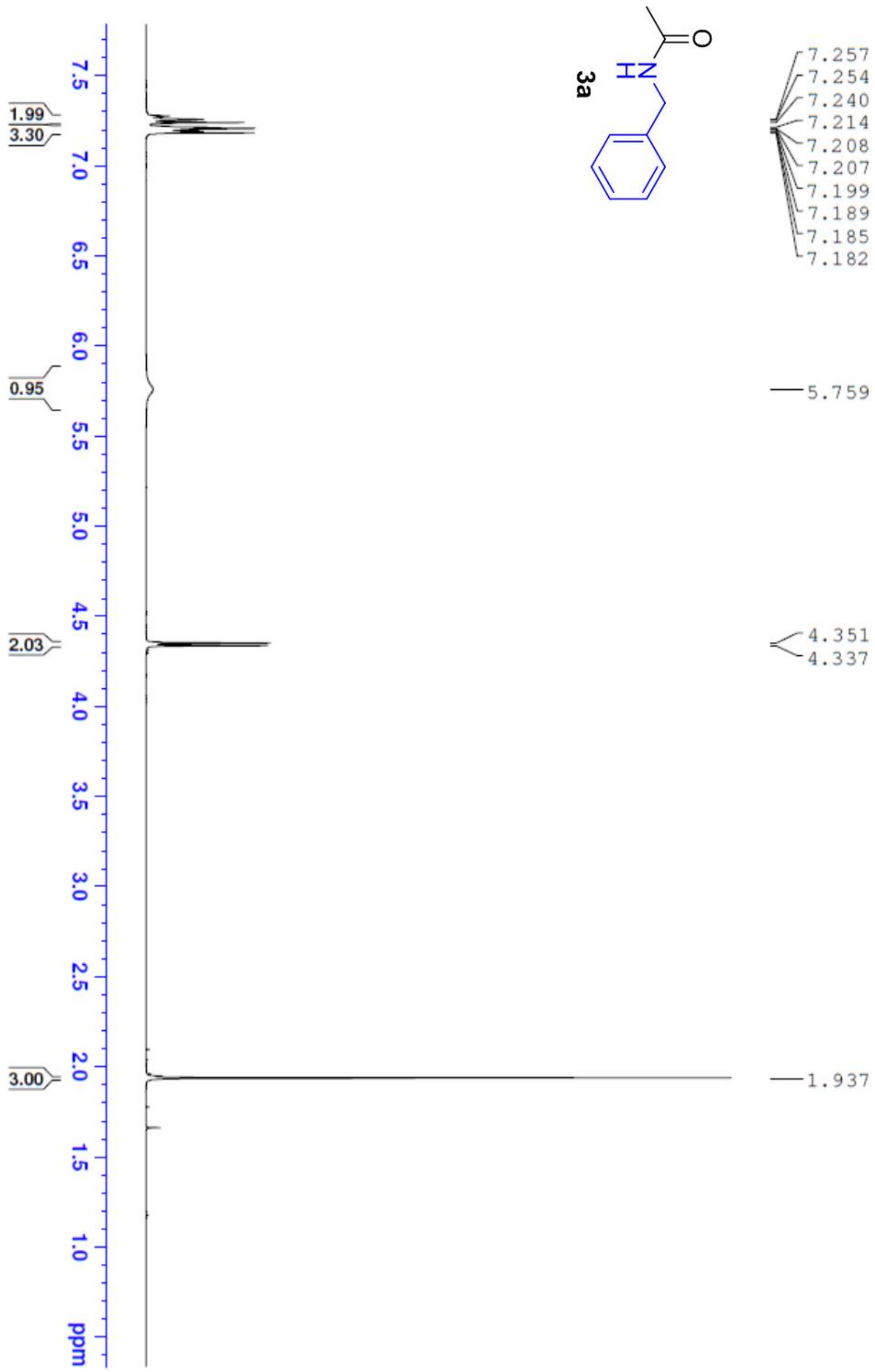

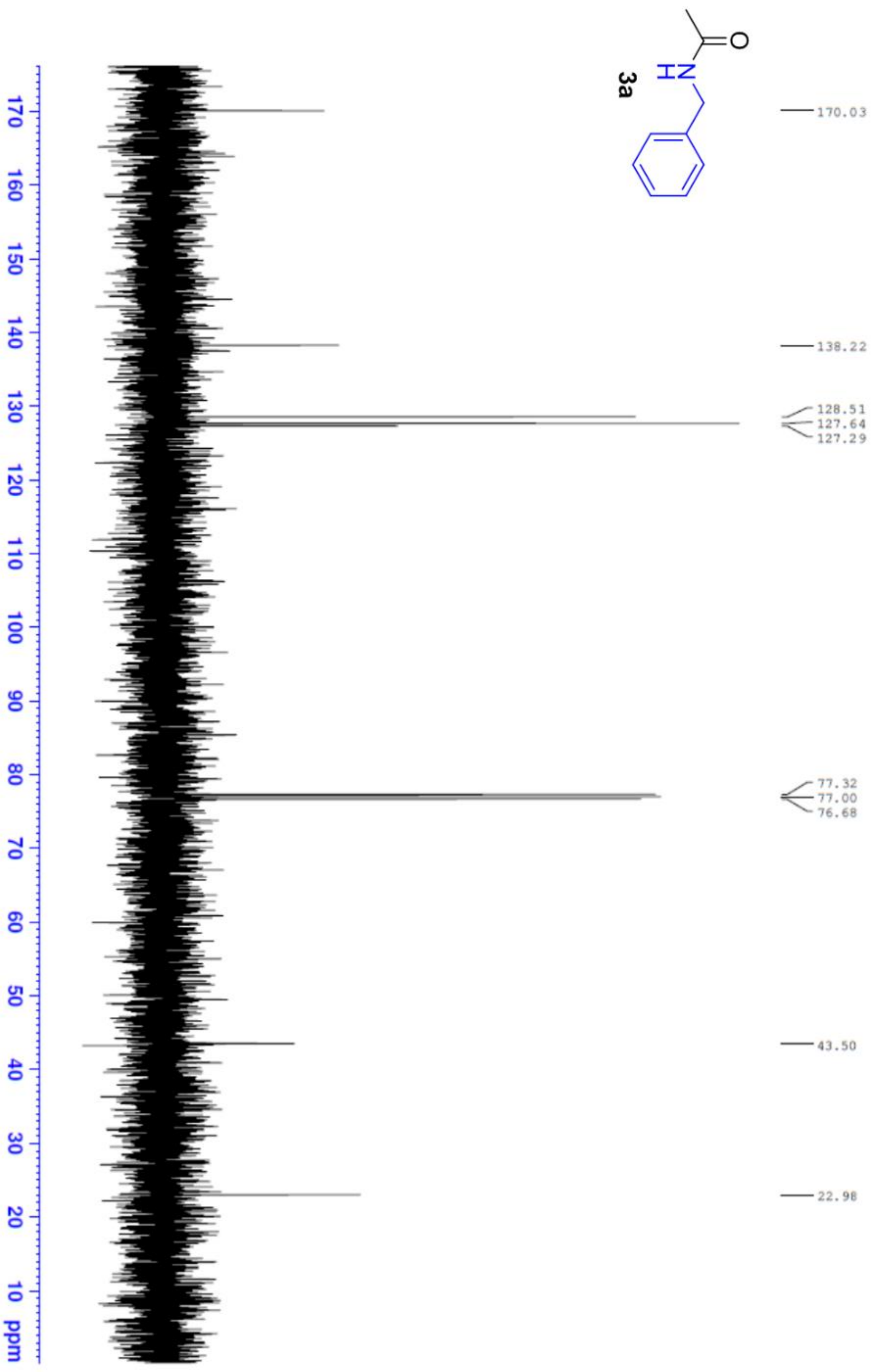
3a
170.03
138.22
128.51
127.64
127.29
77.32
77.00
76.68
43.50
22.98
ppm

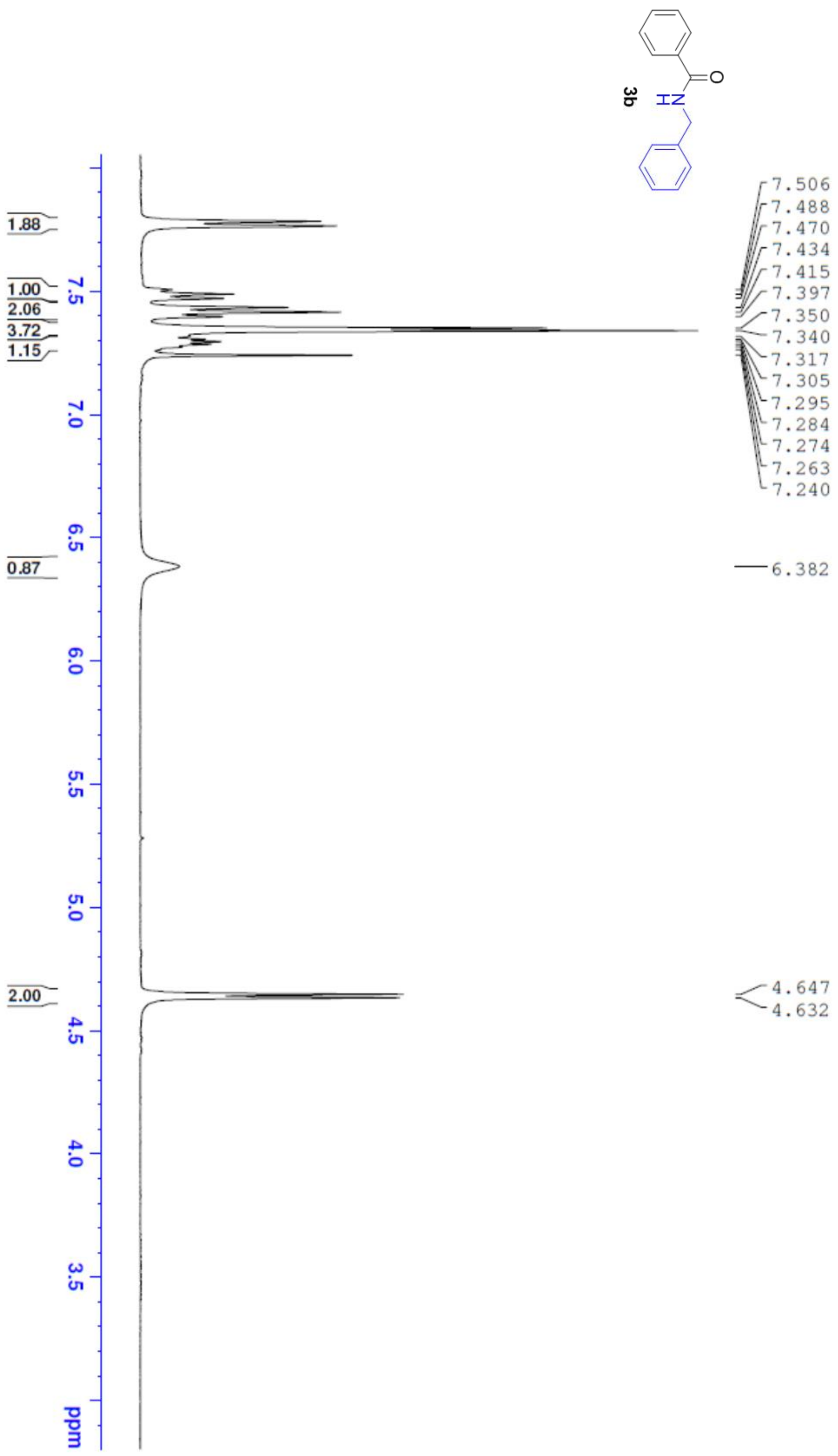

3b
7.506
7.488
7.470
7.434
7.415
7.397
7.350
7.340
7.317
7.305
7.295
7.284
7.274
7.263
7.240
6.382
4.647
4.632
1.88
1.00
2.06
3.72
1.15
0.87
2.00
7.5
7.0
6.5
6.0
5.5
5.0
4.5
4.0
3.5
ppm

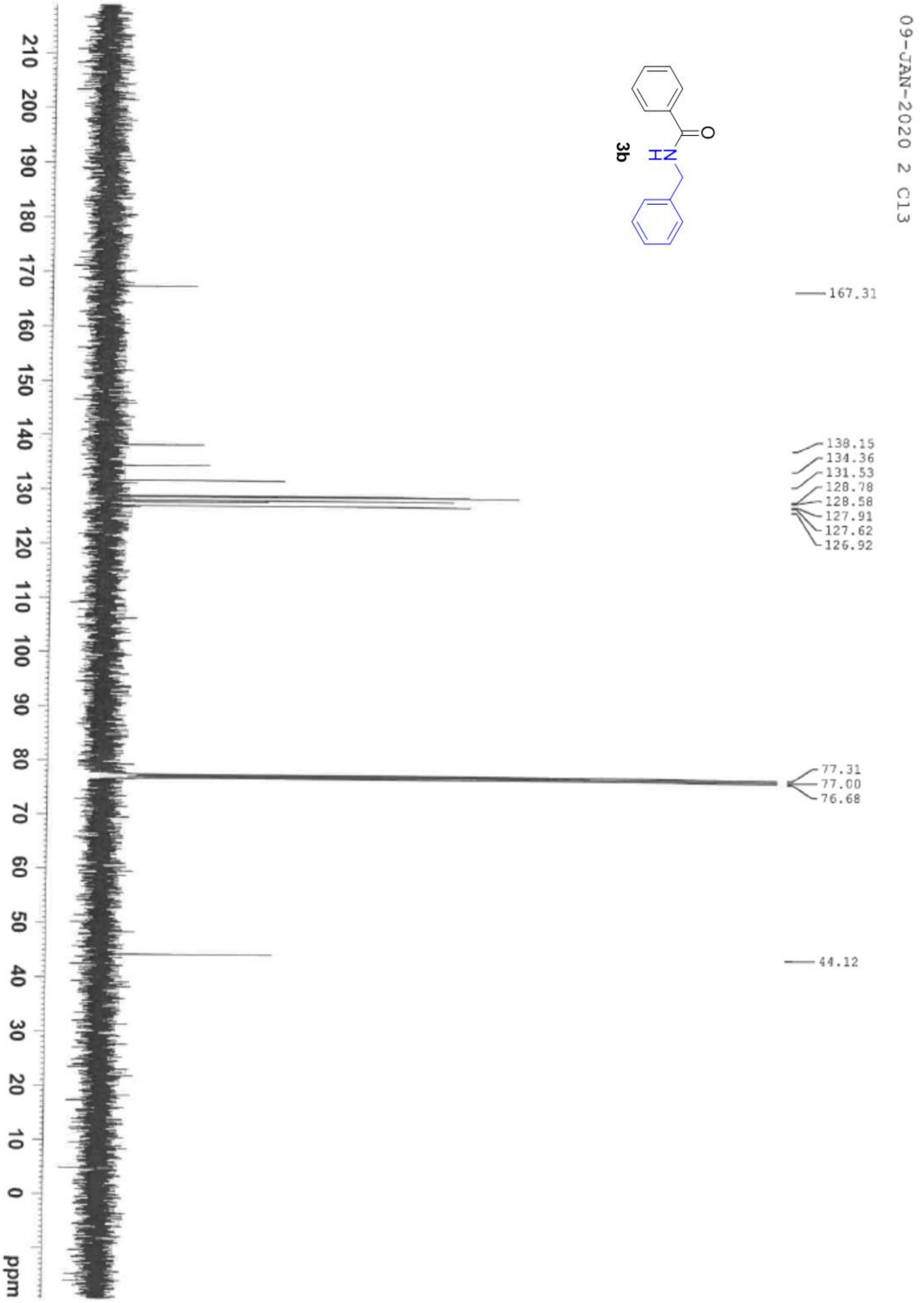

09-JAN-2020 2 C13
O
N
H
3b
167.31
138.15
134.36
131.53
128.78
128.58
127.91
127.62
126.92
77.31
77.00
76.68
44.12
210 200 190 180 170 160 150 140 130 120 110 100 90 80 70 60 50 40 30 20 10 0 ppm

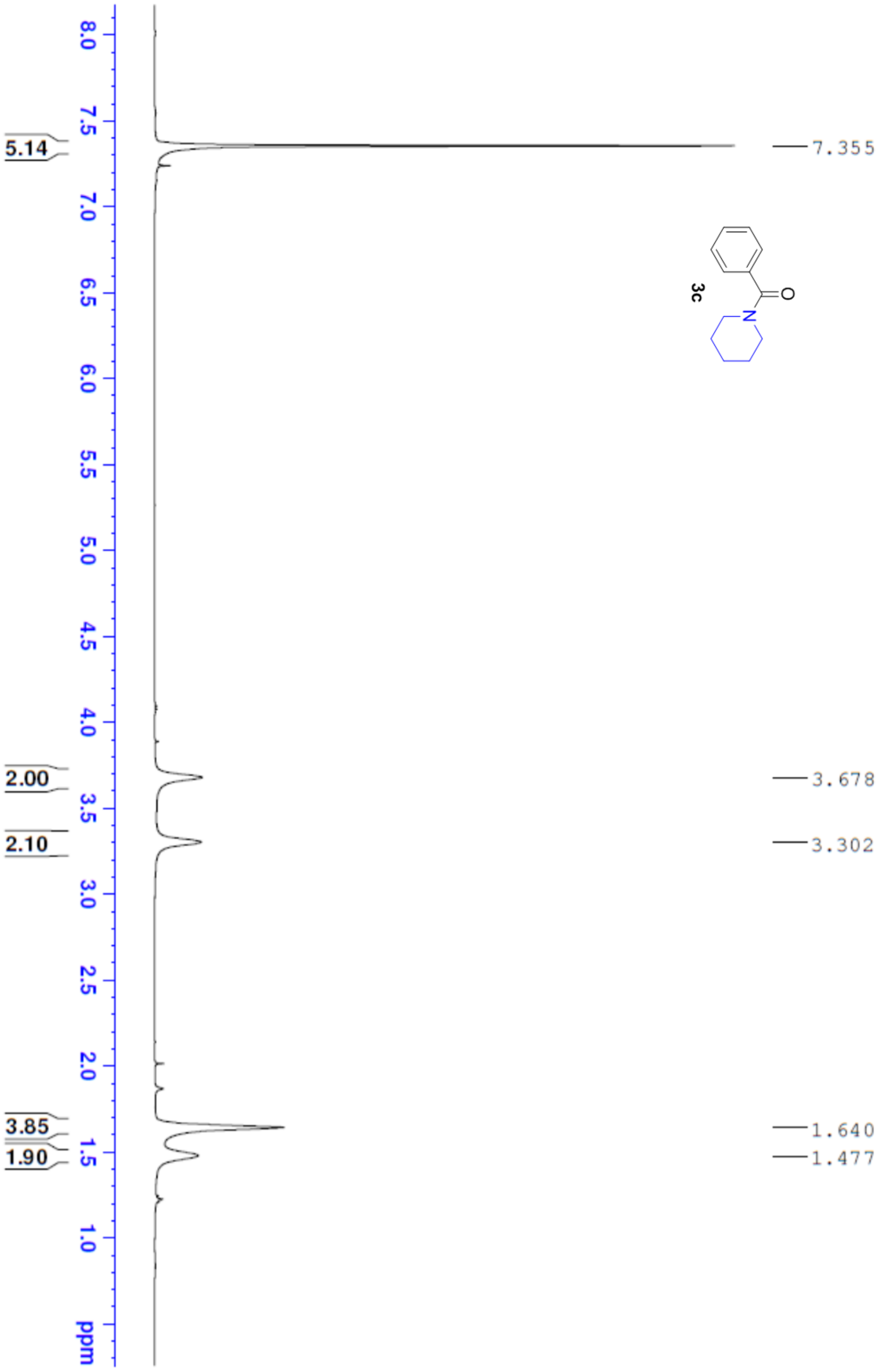
7.355
3.678
3.302
1.640
1.477
5.14
2.00
2.10
3.85
1.90
3c
ppm

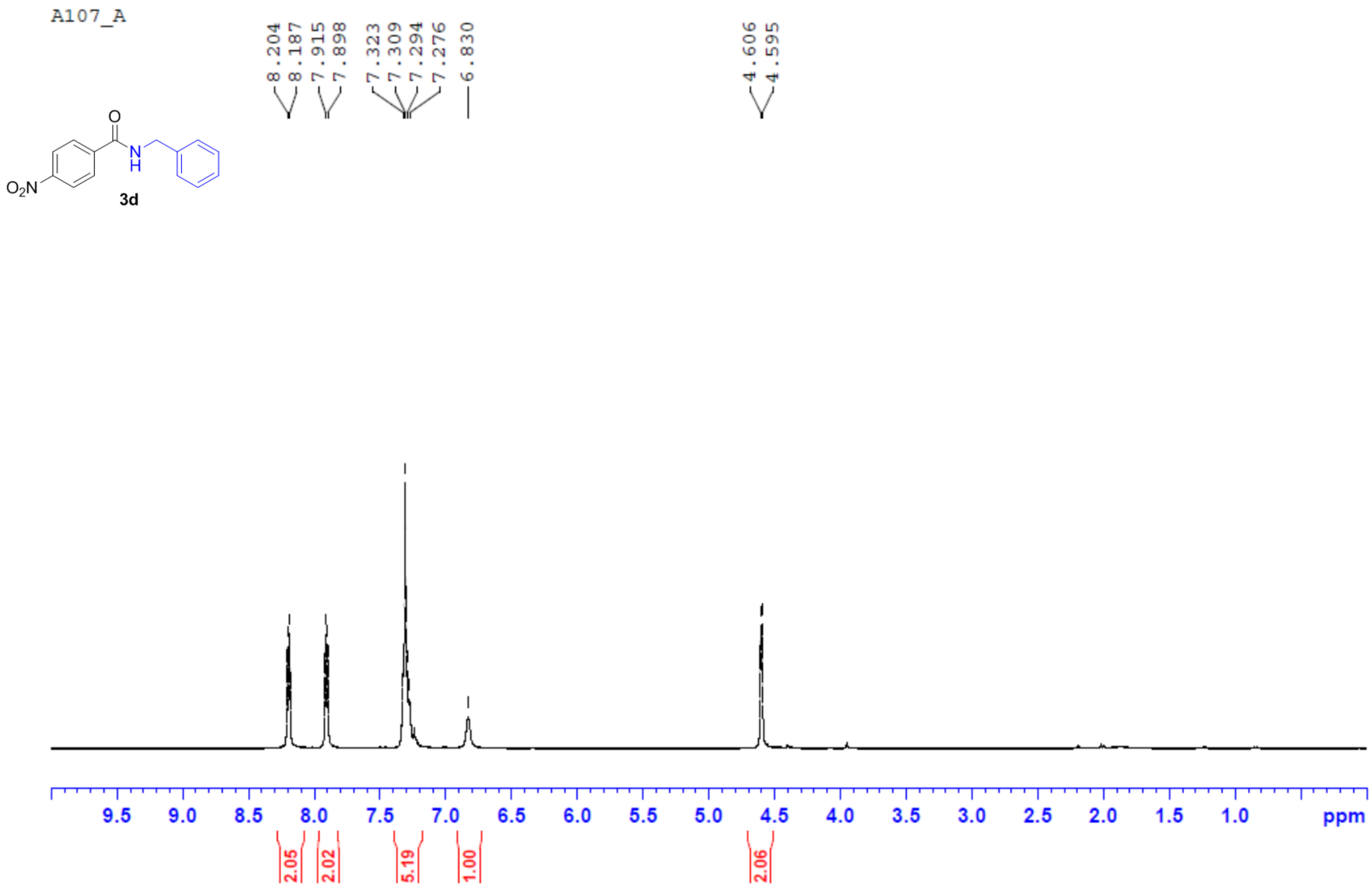
A107_A
8.204
8.187
7.915
7.898
7.323
7.309
7.294
7.276
6.830
4.606
4.595
O
N
H
O2N
3d
9.5
9.0
8.5
8.0
7.5
7.0
6.5
6.0
5.5
5.0
4.5
4.0
3.5
3.0
2.5
2.0
1.5
1.0
ppm
2.05
2.02
5.19
1.00
2.06

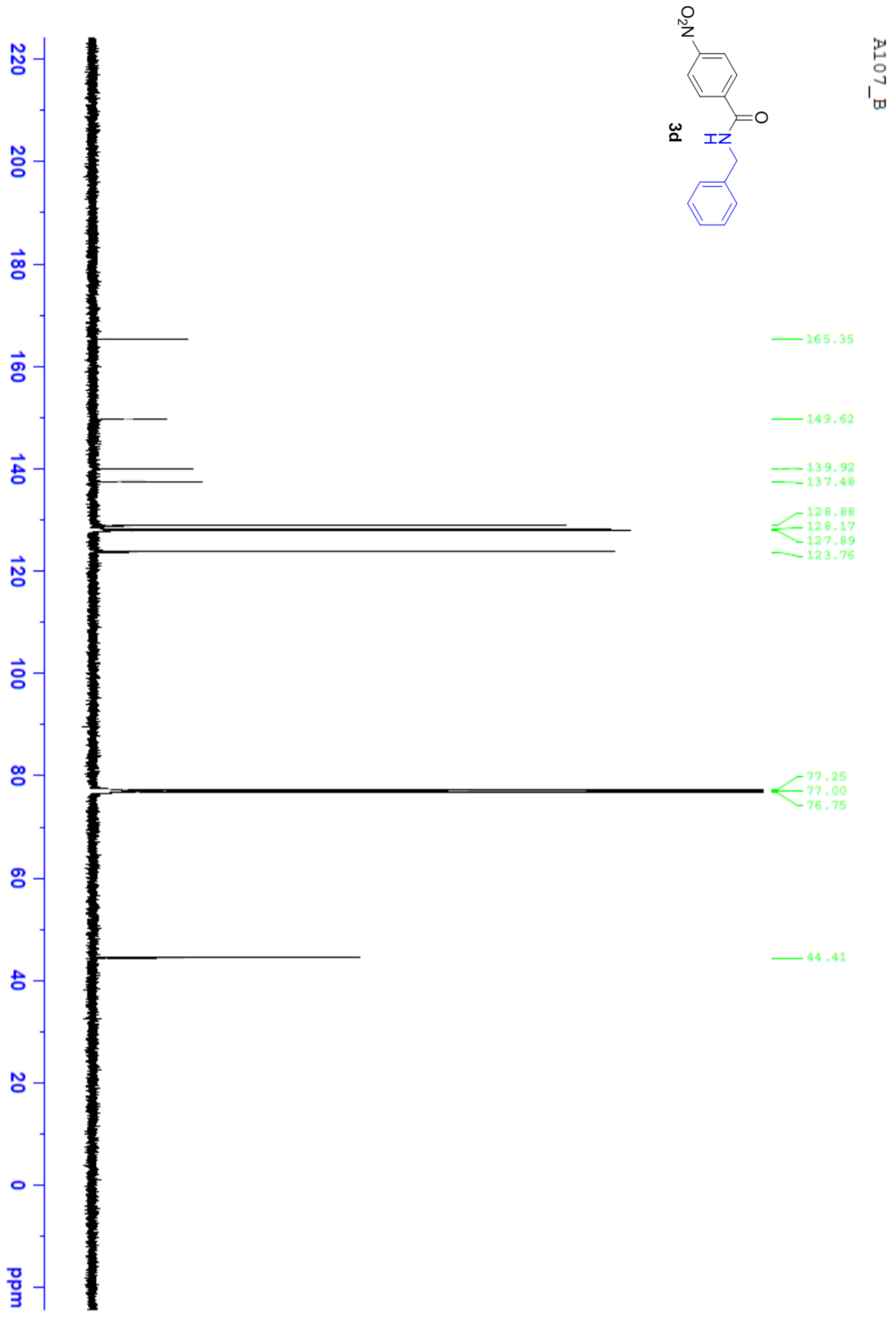

A107_B
3d
165.35
149.62
139.92
137.48
128.88
128.17
127.89
123.76
77.25
77.00
76.75
44.41
220
200
180
160
140
120
100
80
60
40
20
0
ppm

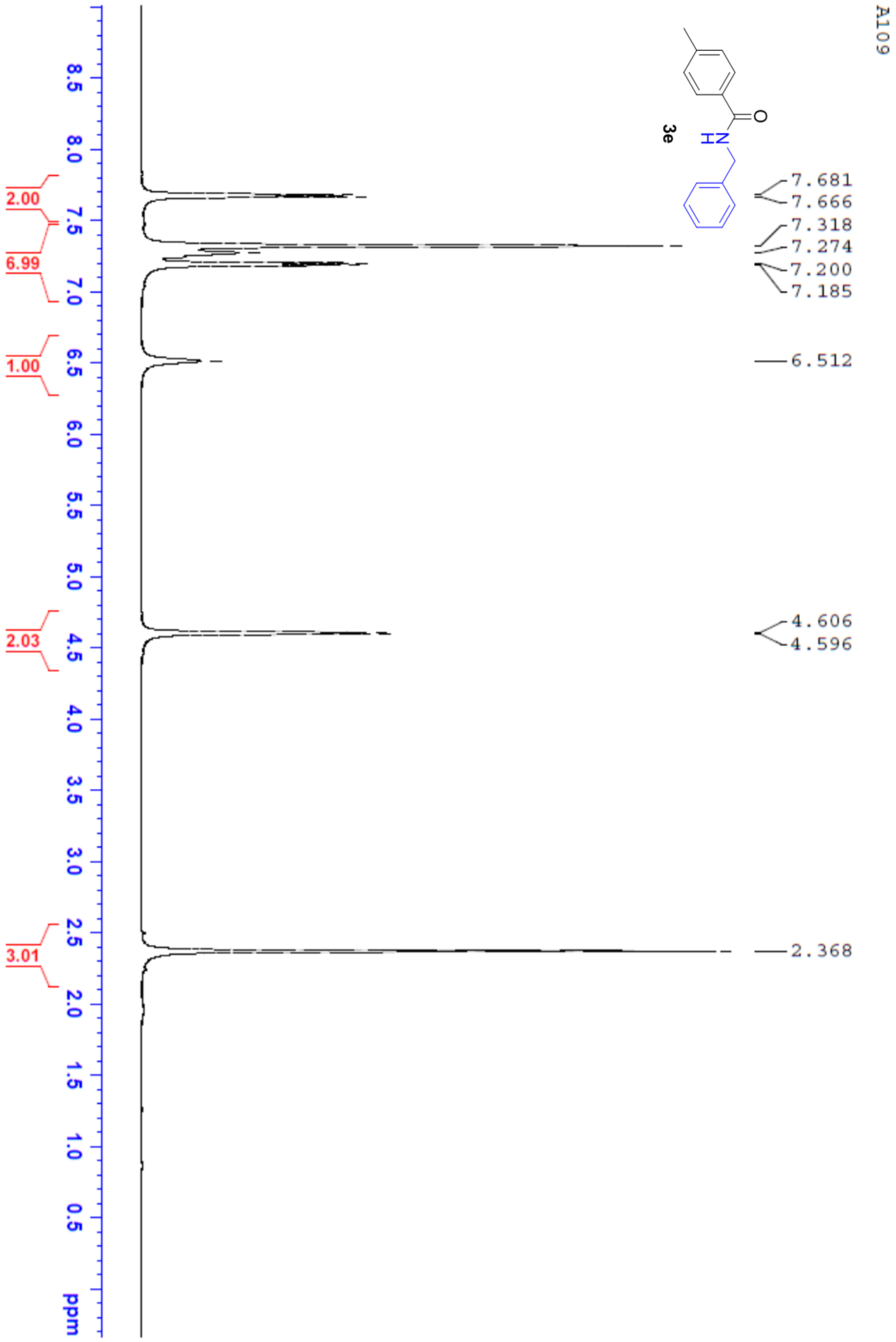
A109
3e
7.681
7.666
7.318
7.274
7.200
7.185
6.512
4.606
4.596
2.368
2.00
6.99
1.00
2.03
3.01
ppm

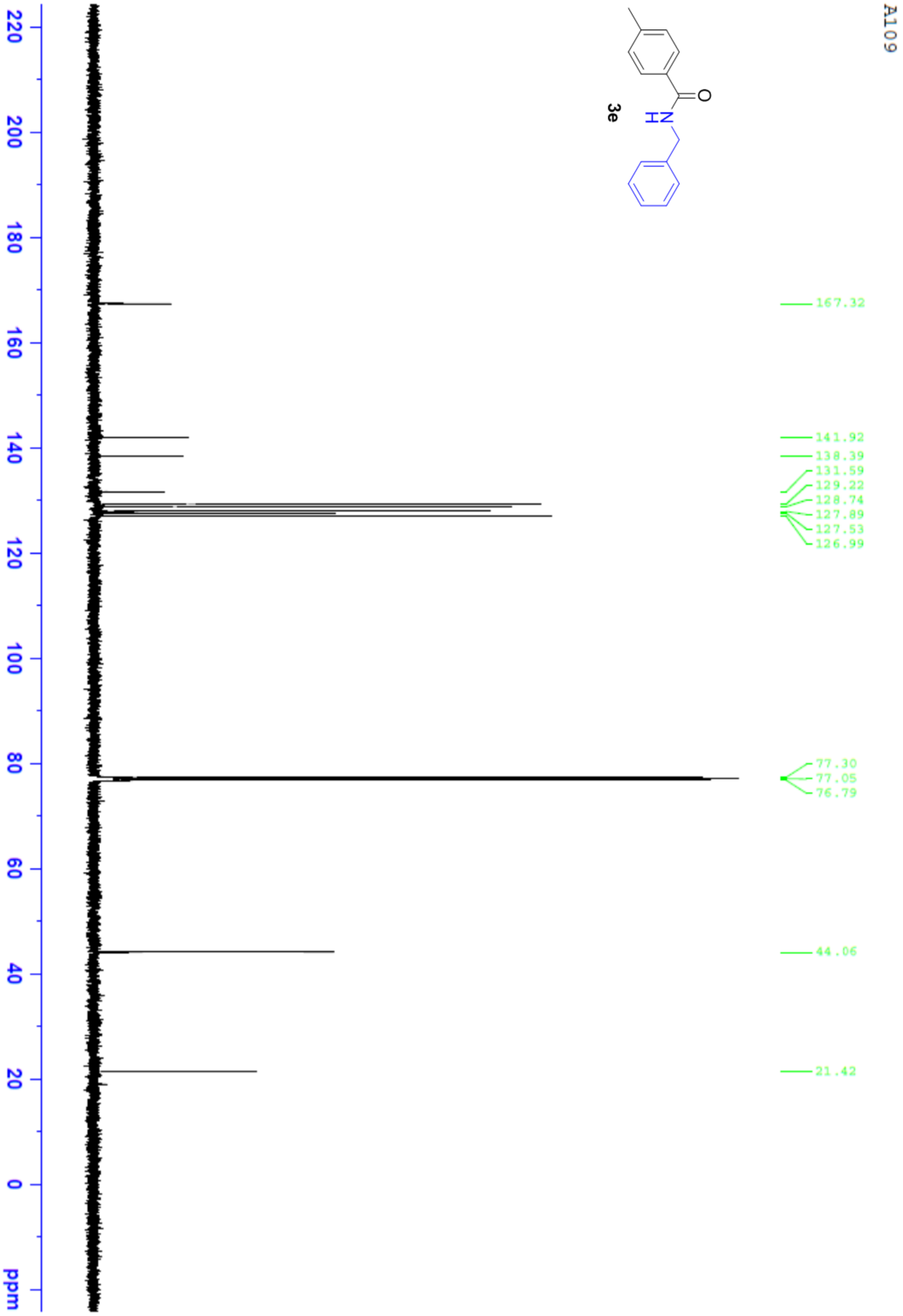
A109
3e
167.32
141.92
138.39
131.59
129.22
128.74
127.89
127.53
126.99
77.30
77.05
76.79
44.06
21.42
220
200
180
160
140
120
100
80
60
40
20
0
ppm

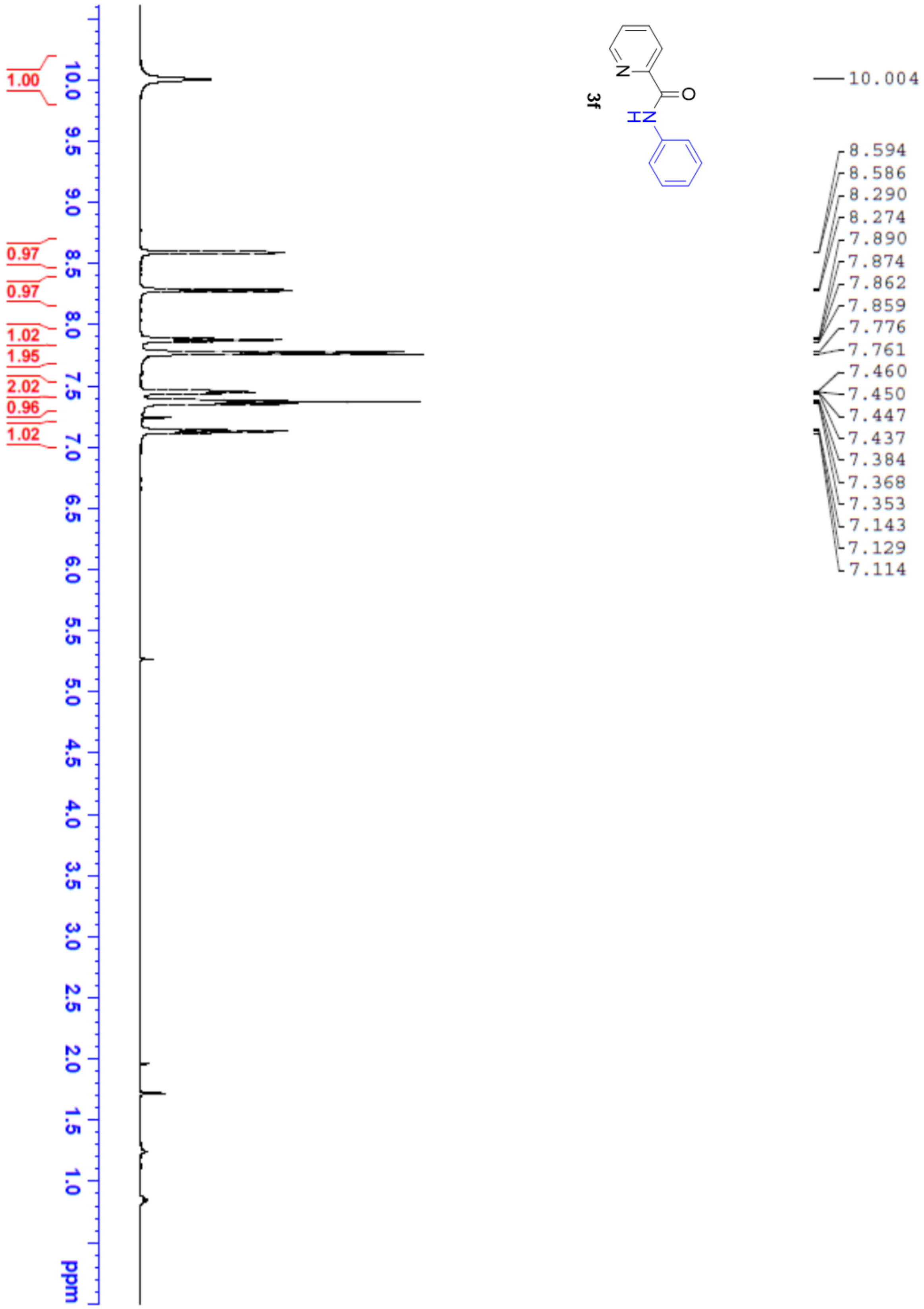
3f
10.004
8.594
8.586
8.290
8.274
7.890
7.874
7.862
7.859
7.776
7.761
7.460
7.450
7.447
7.437
7.384
7.368
7.353
7.143
7.129
7.114
1.00
0.97
0.97
1.02
1.95
2.02
0.96
1.02
ppm

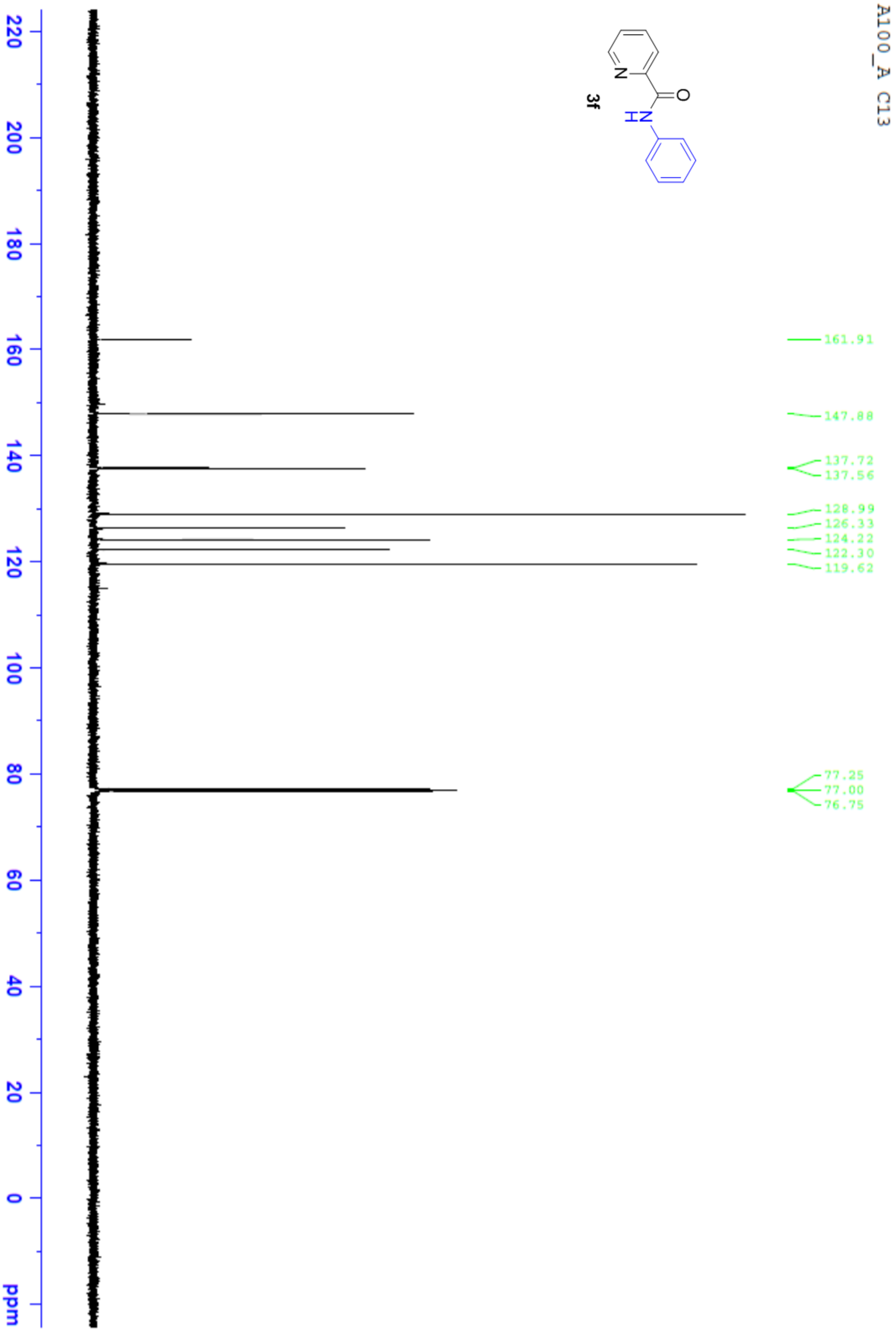

A100_A C13
3f
161.91
147.88
137.72
137.56
128.99
126.33
124.22
122.30
119.62
77.25
77.00
76.75
220
200
180
160
140
120
100
80
60
40
20
0
ppm

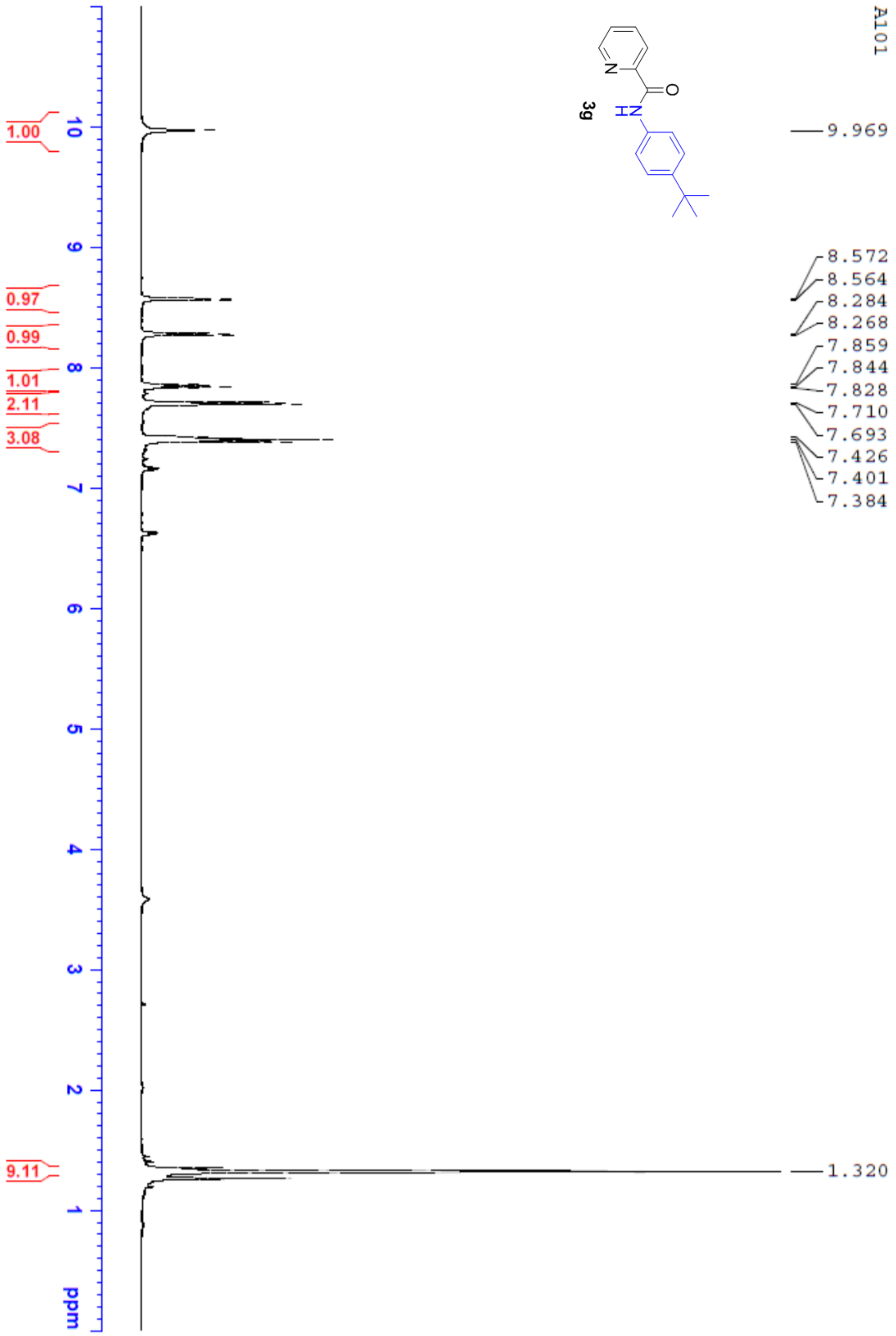
A101
3g
9.969
8.572
8.564
8.284
8.268
7.859
7.844
7.828
7.710
7.693
7.426
7.401
7.384
1.320
1.00
0.97
0.99
1.01
2.11
3.08
9.11
ppm

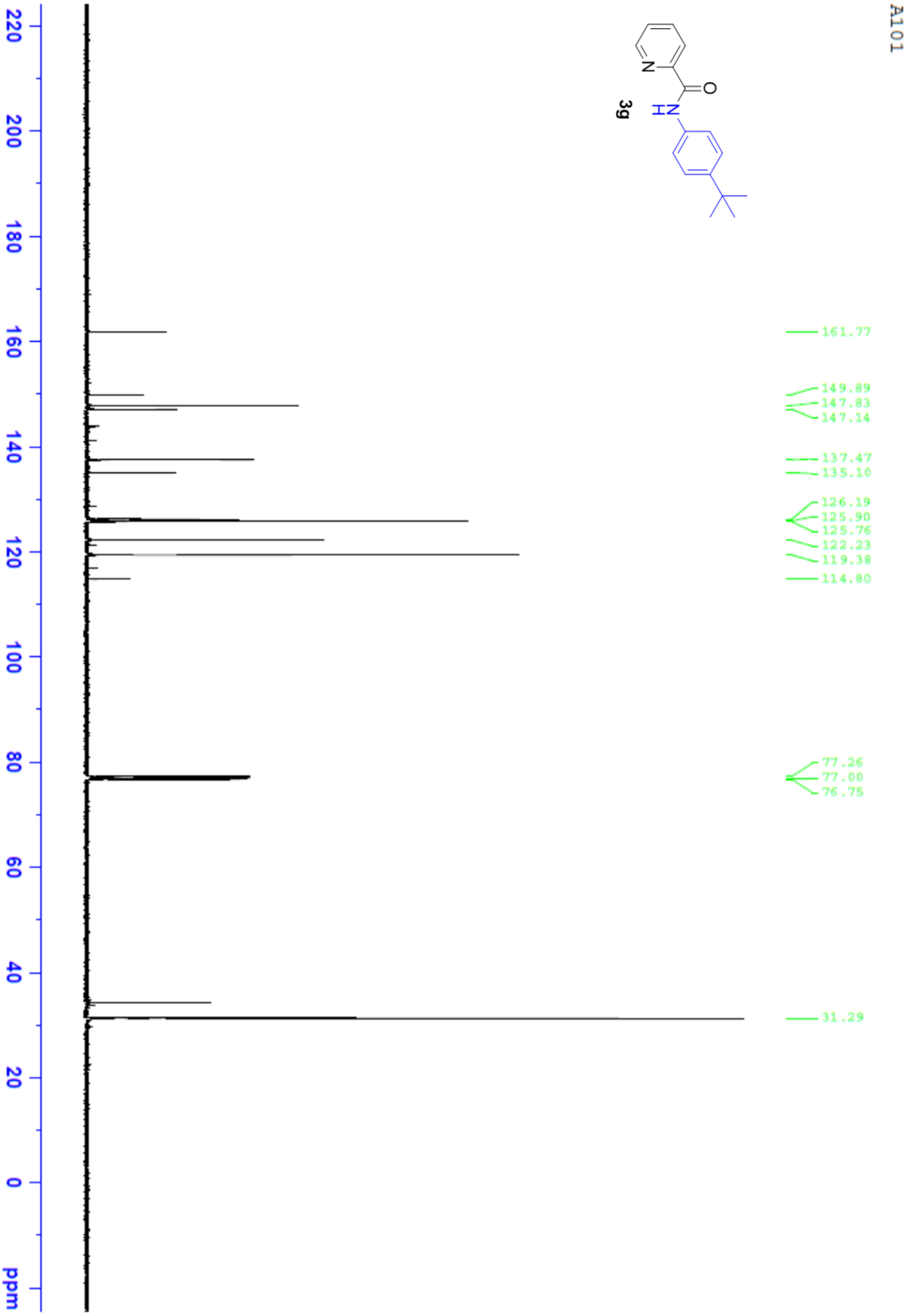
A101
3g
161.77
149.89
147.83
147.14
137.47
135.10
126.19
125.90
125.76
122.23
119.38
114.80
77.26
77.00
76.75
31.29
220
200
180
160
140
120
100
80
60
40
20
0
ppm

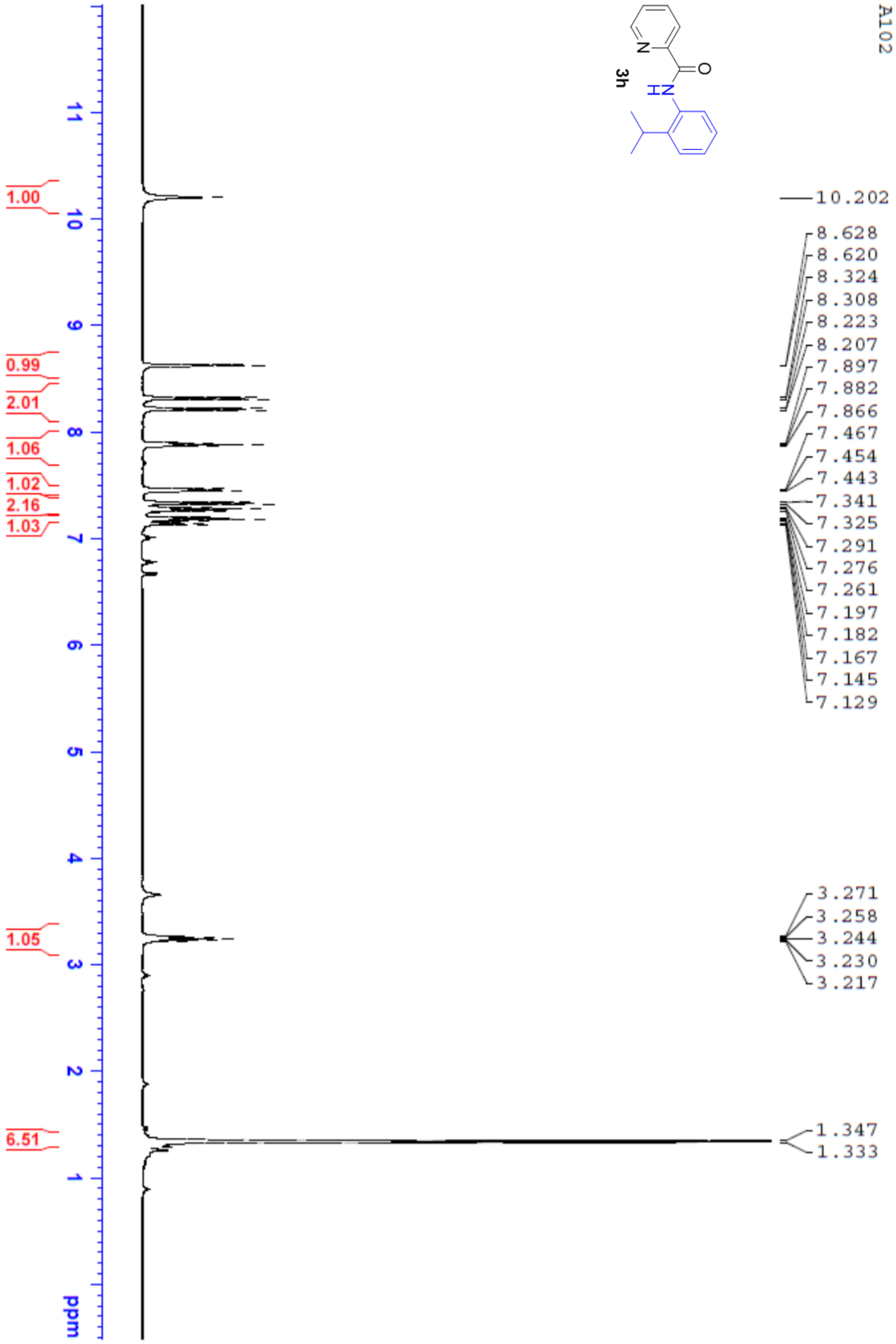
A102
3h
10.202
8.628
8.620
8.324
8.308
8.223
8.207
7.897
7.882
7.866
7.467
7.454
7.443
7.341
7.325
7.291
7.276
7.261
7.197
7.182
7.167
7.145
7.129
3.271
3.258
3.244
3.230
3.217
1.347
1.333
1.00
0.99
2.01
1.06
1.02
2.16
1.03
1.05
6.51
ppm

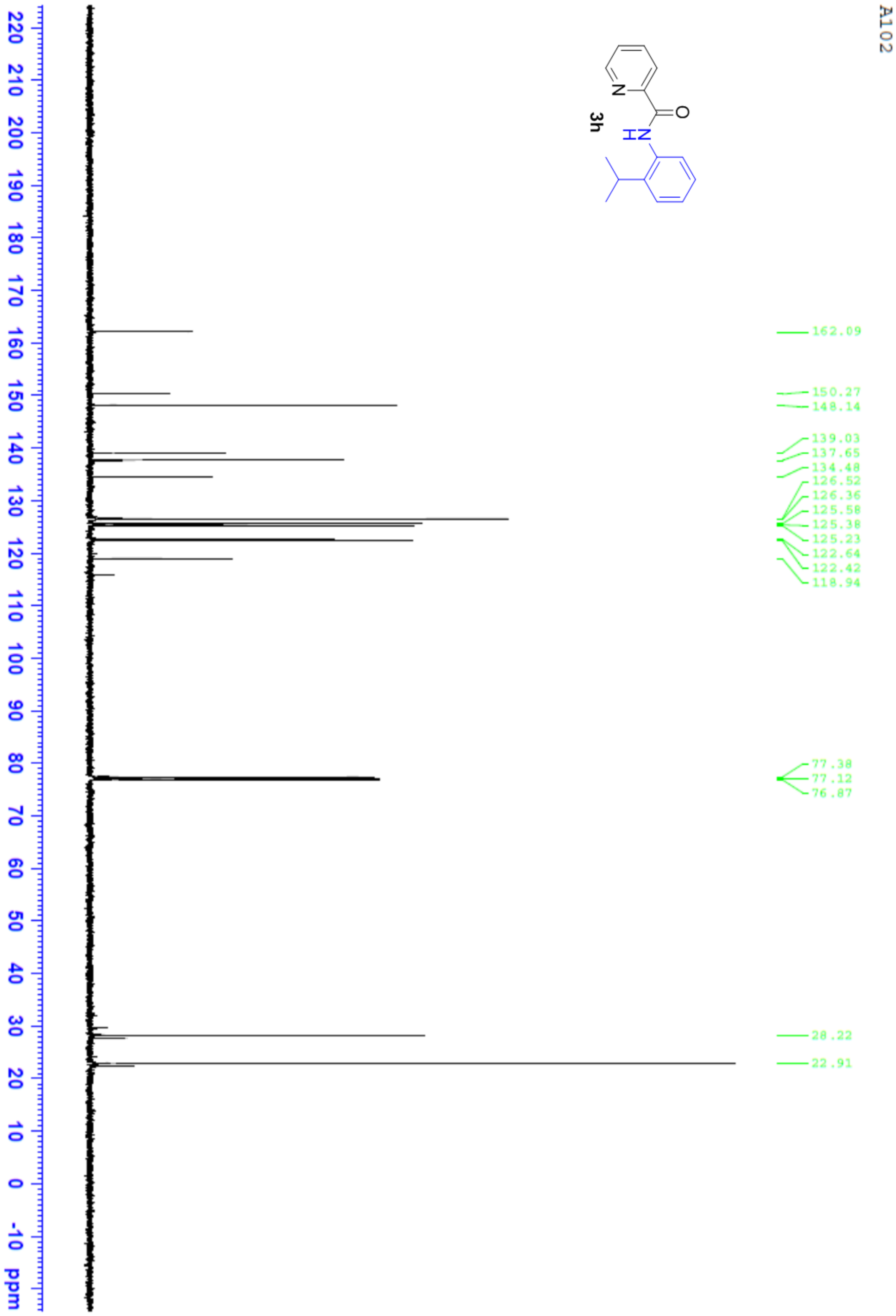
A102
3h
162.09
150.27
148.14
139.03
137.65
134.48
126.52
126.36
125.58
125.38
125.23
122.64
122.42
118.94
77.38
77.12
76.87
28.22
22.91
220 210 200 190 180 170 160 150 140 130 120 110 100 90 80 70 60 50 40 30 20 10 0 -10 ppm

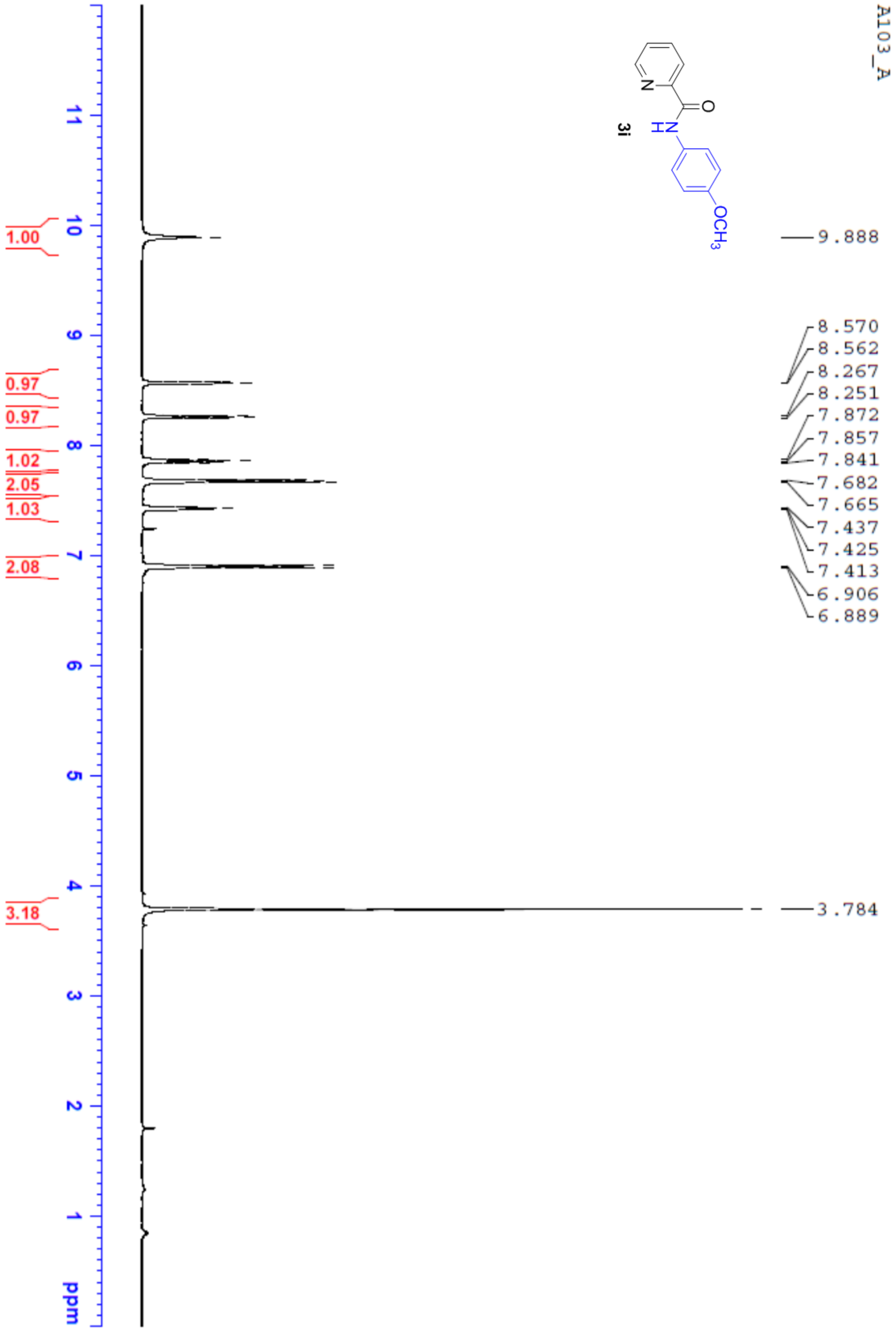
A103_A
3i
OCH3
9.888
8.570
8.562
8.267
8.251
7.872
7.857
7.841
7.682
7.665
7.437
7.425
7.413
6.906
6.889
3.784
1.00
0.97
0.97
1.02
2.05
1.03
2.08
3.18
ppm

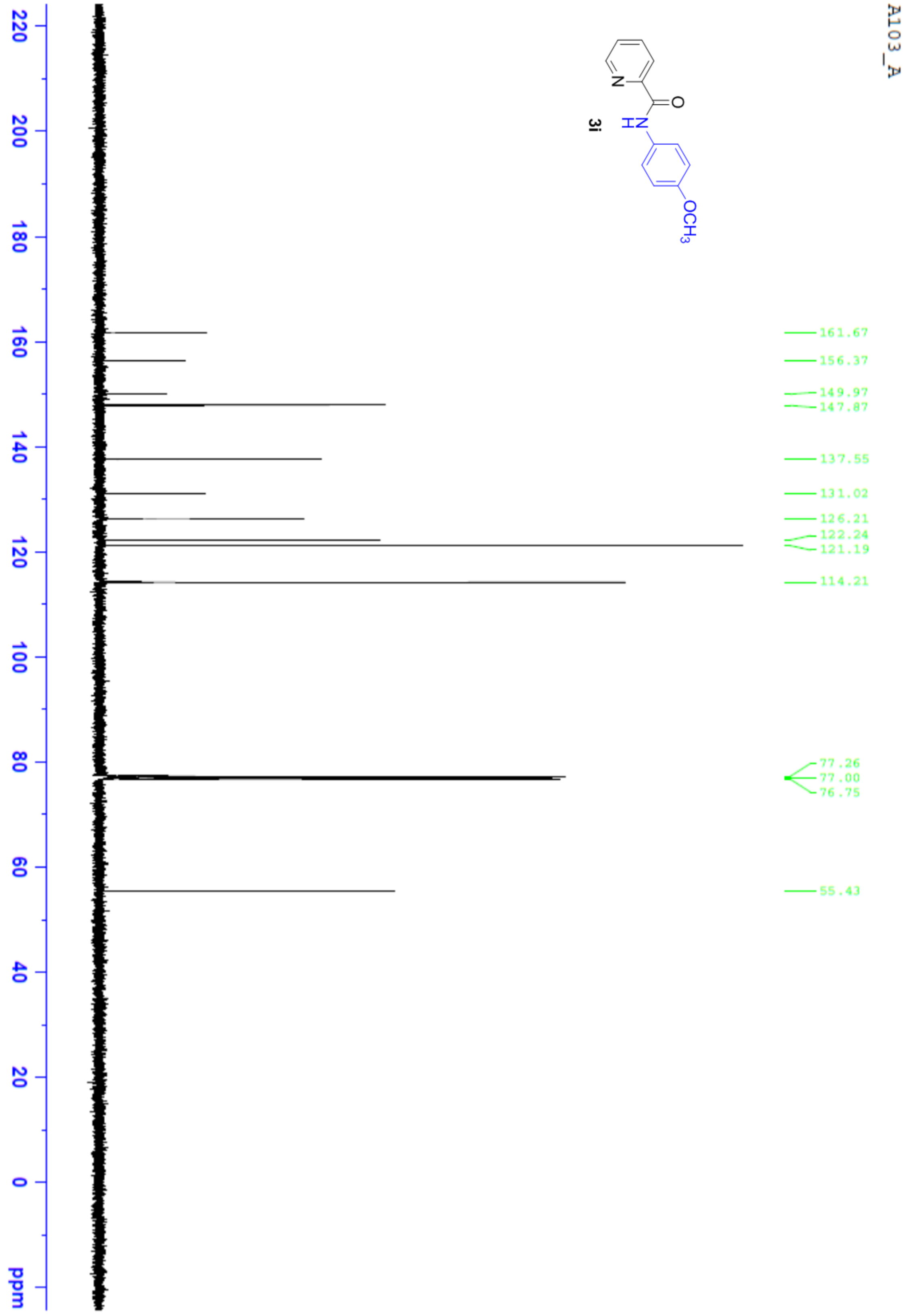
A103_A
3i
OCH$_3$
161.67
156.37
149.97
147.87
137.55
131.02
126.21
122.24
121.19
114.21
77.26
77.00
76.75
55.43
220
200
180
160
140
120
100
80
60
40
20
0
ppm

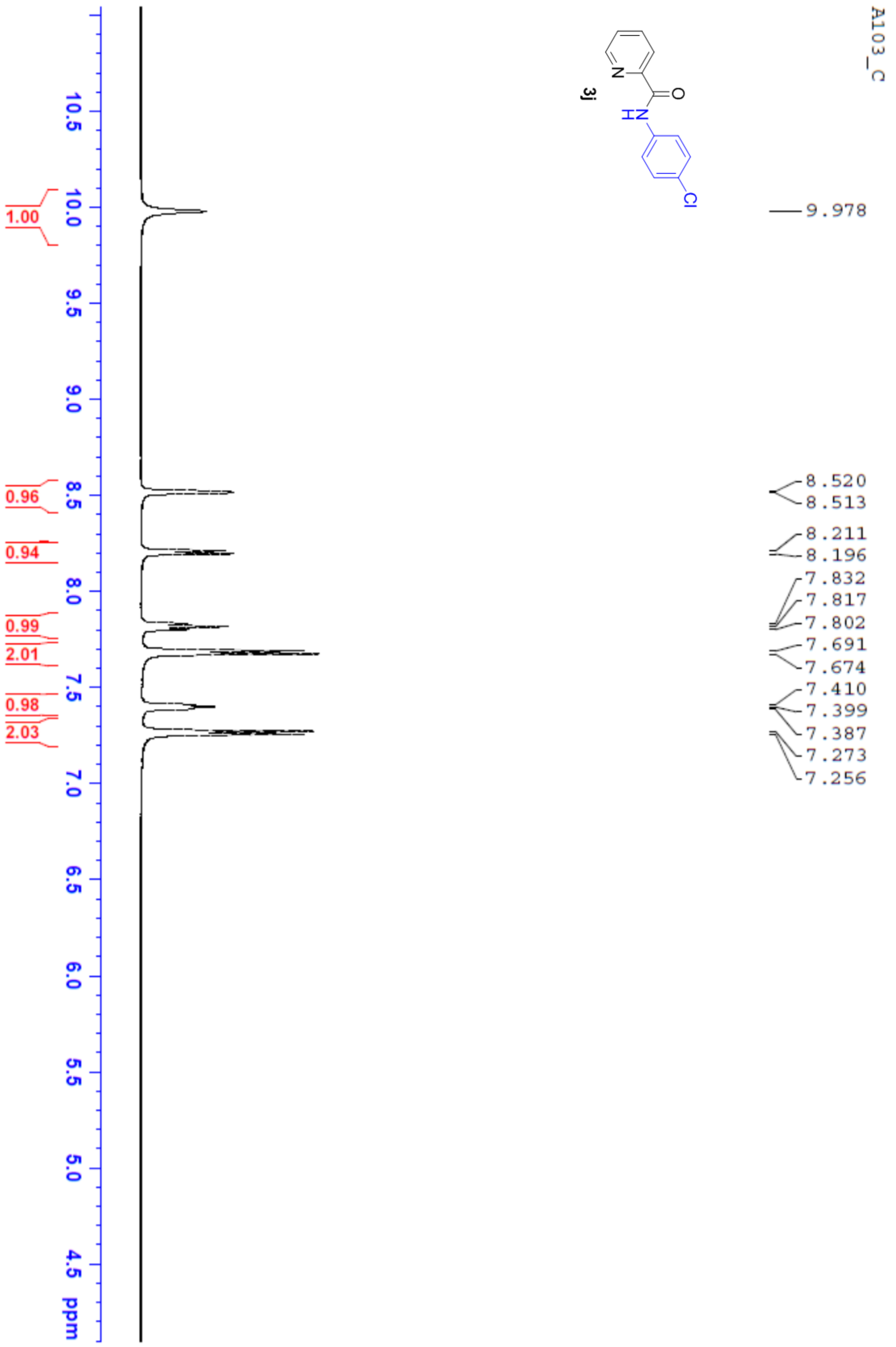
A103_C
3j
N
O
N
H
Cl
9.978
8.520
8.513
8.211
8.196
7.832
7.817
7.802
7.691
7.674
7.410
7.399
7.387
7.273
7.256
1.00
0.96
0.94
0.99
2.01
0.98
2.03
10.5
10.0
9.5
9.0
8.5
8.0
7.5
7.0
6.5
6.0
5.5
5.0
4.5
ppm

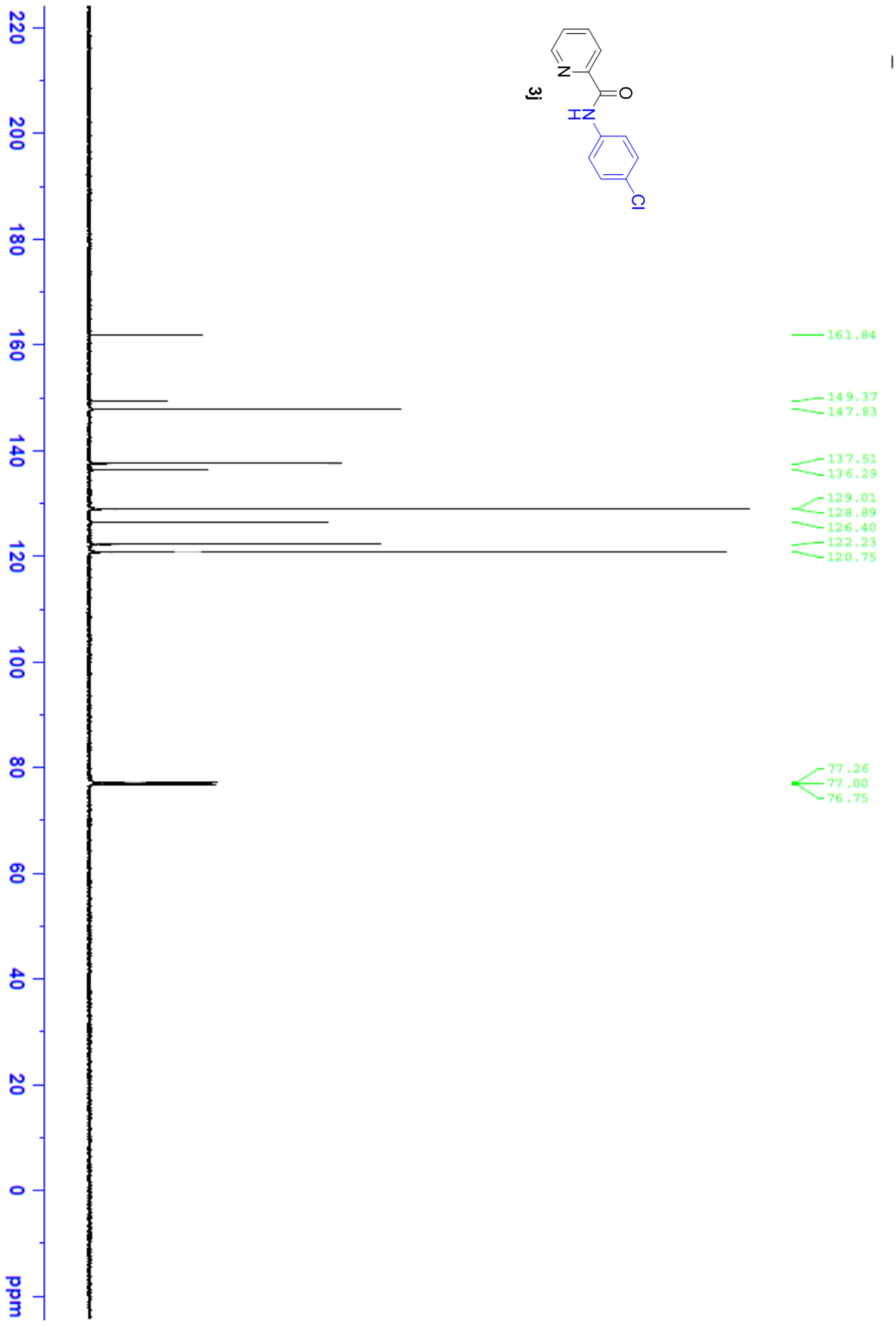
A103_C
3j
161.84
149.37
147.83
137.51
136.29
129.01
128.89
126.40
122.23
120.75
77.26
77.00
76.75
220
200
180
160
140
120
100
80
60
40
20
0
ppm

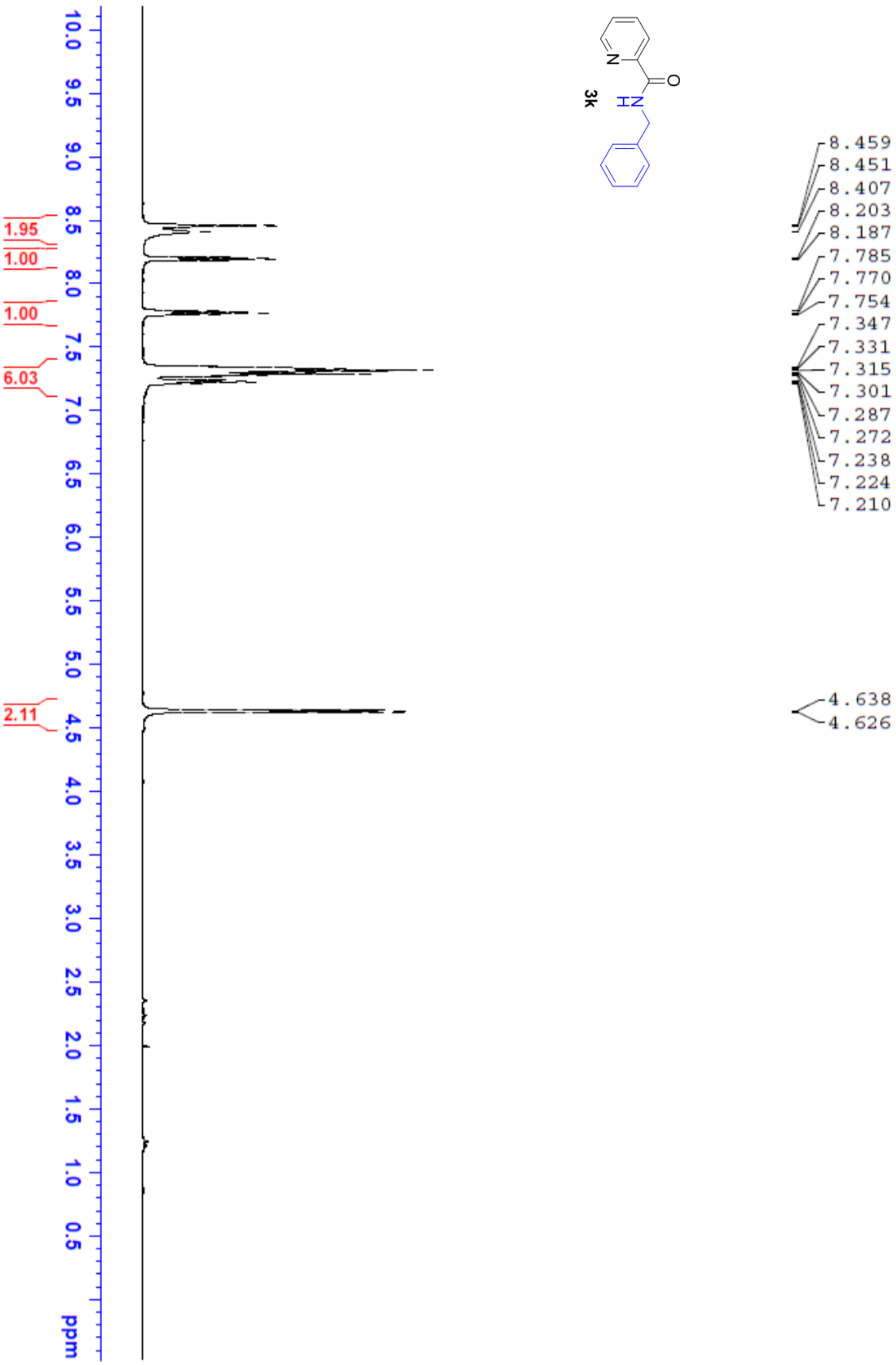
3k
8.459
8.451
8.407
8.203
8.187
7.785
7.770
7.754
7.347
7.331
7.315
7.301
7.287
7.272
7.238
7.224
7.210
4.638
4.626
1.95
1.00
1.00
6.03
2.11
10.0
9.5
9.0
8.5
8.0
7.5
7.0
6.5
6.0
5.5
5.0
4.5
4.0
3.5
3.0
2.5
2.0
1.5
1.0
0.5
ppm

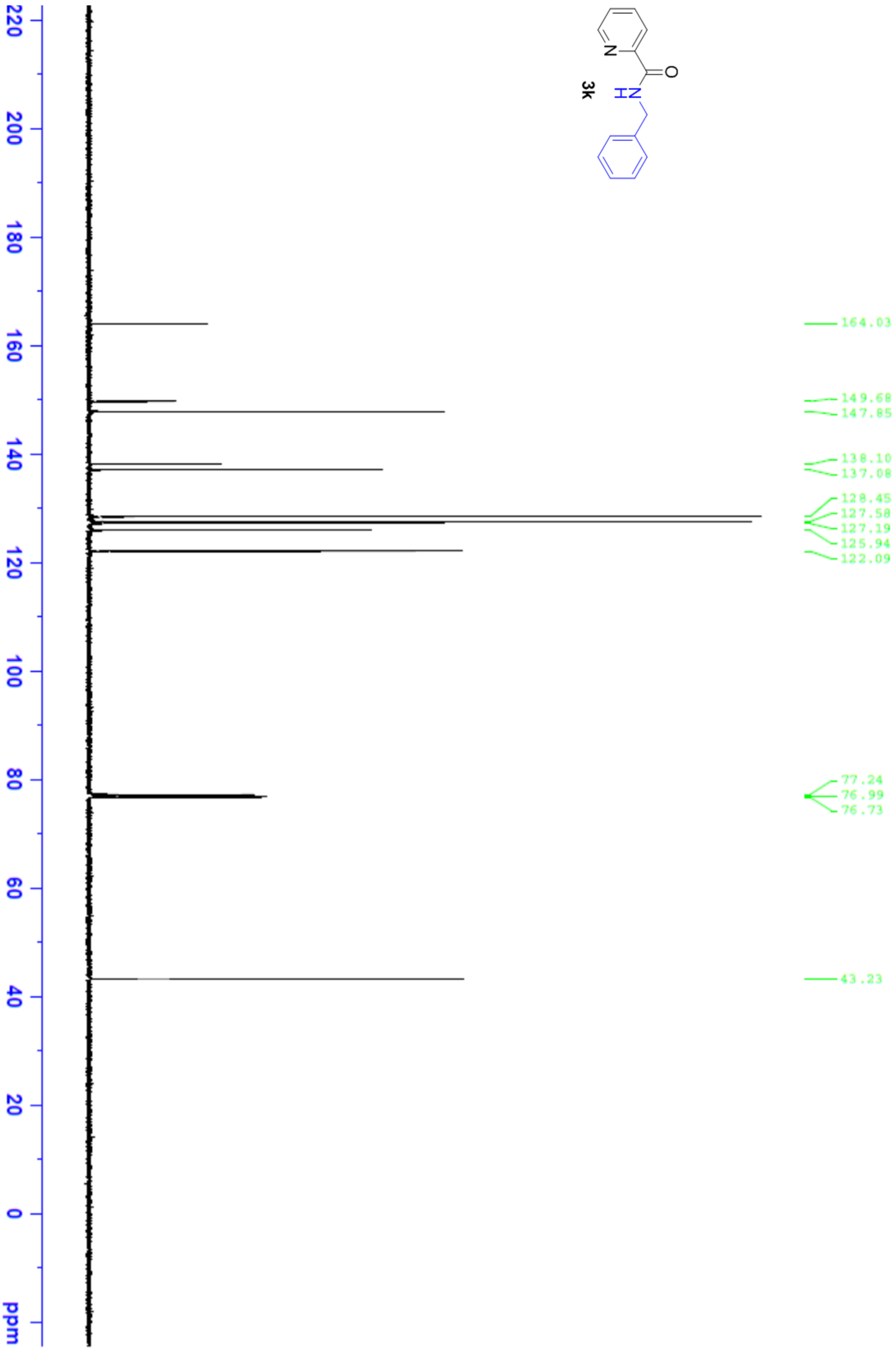
3k
164.03
149.68
147.85
138.10
137.08
128.45
127.58
127.19
125.94
122.09
77.24
76.99
76.73
43.23
ppm

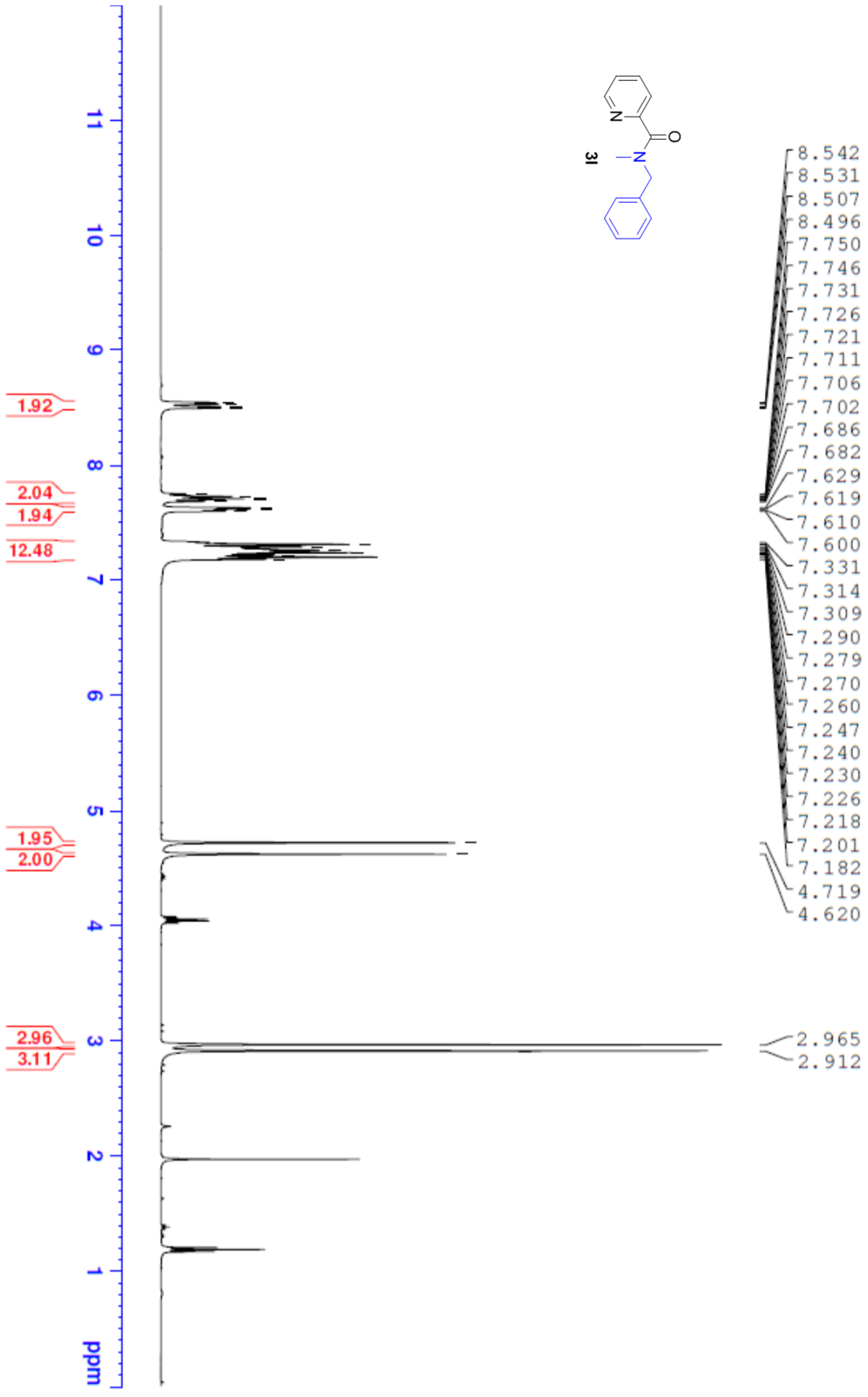
3l
8.542
8.531
8.507
8.496
7.750
7.746
7.731
7.726
7.721
7.711
7.706
7.702
7.686
7.682
7.629
7.619
7.610
7.600
7.331
7.314
7.309
7.290
7.279
7.270
7.260
7.247
7.240
7.230
7.226
7.218
7.201
7.182
4.719
4.620
2.965
2.912
1.92
2.04
1.94
12.48
1.95
2.00
2.96
3.11
11
10
9
8
7
6
5
4
3
2
1
ppm

Aug15-2019-RT exp 6 13c

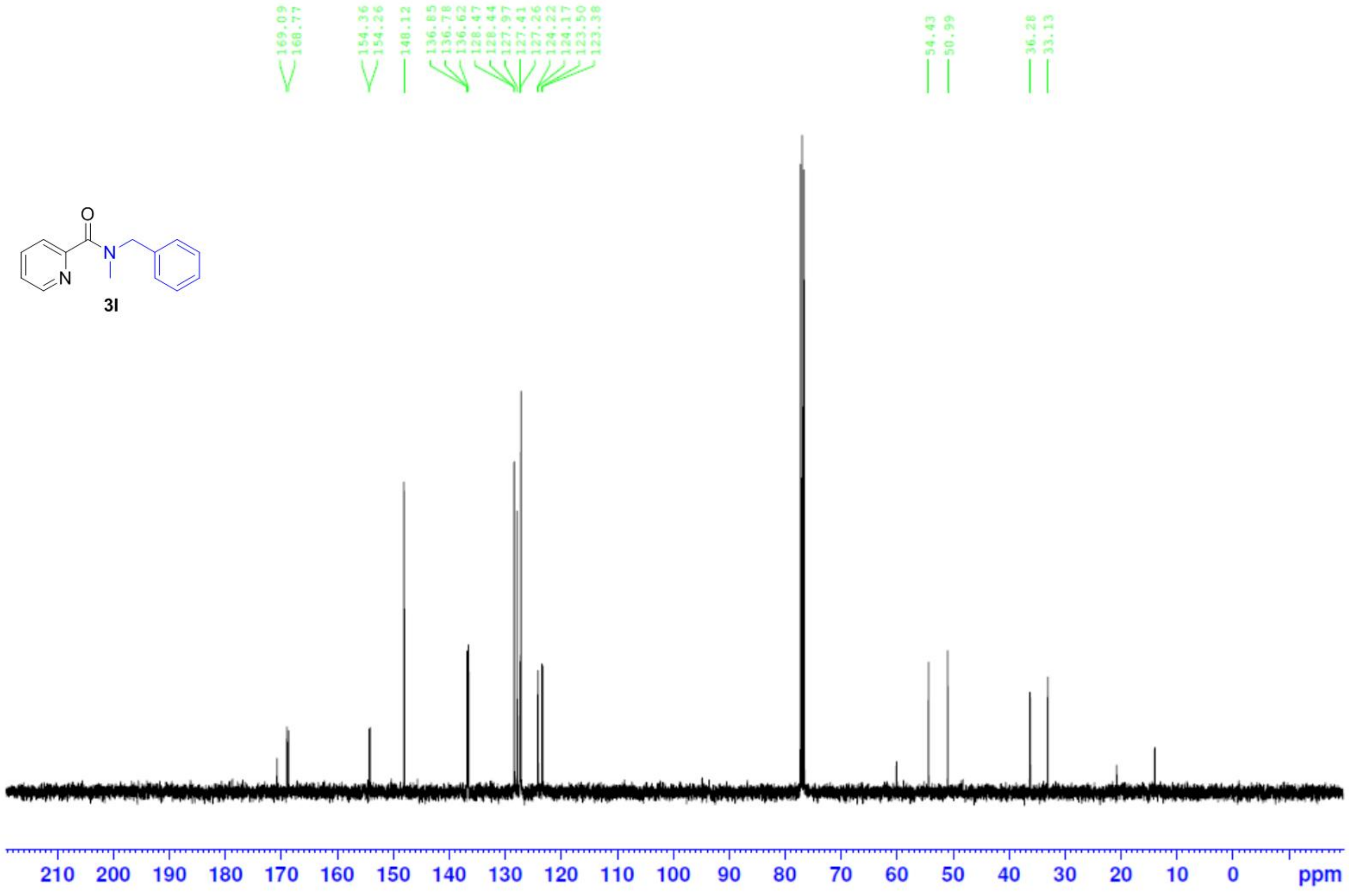

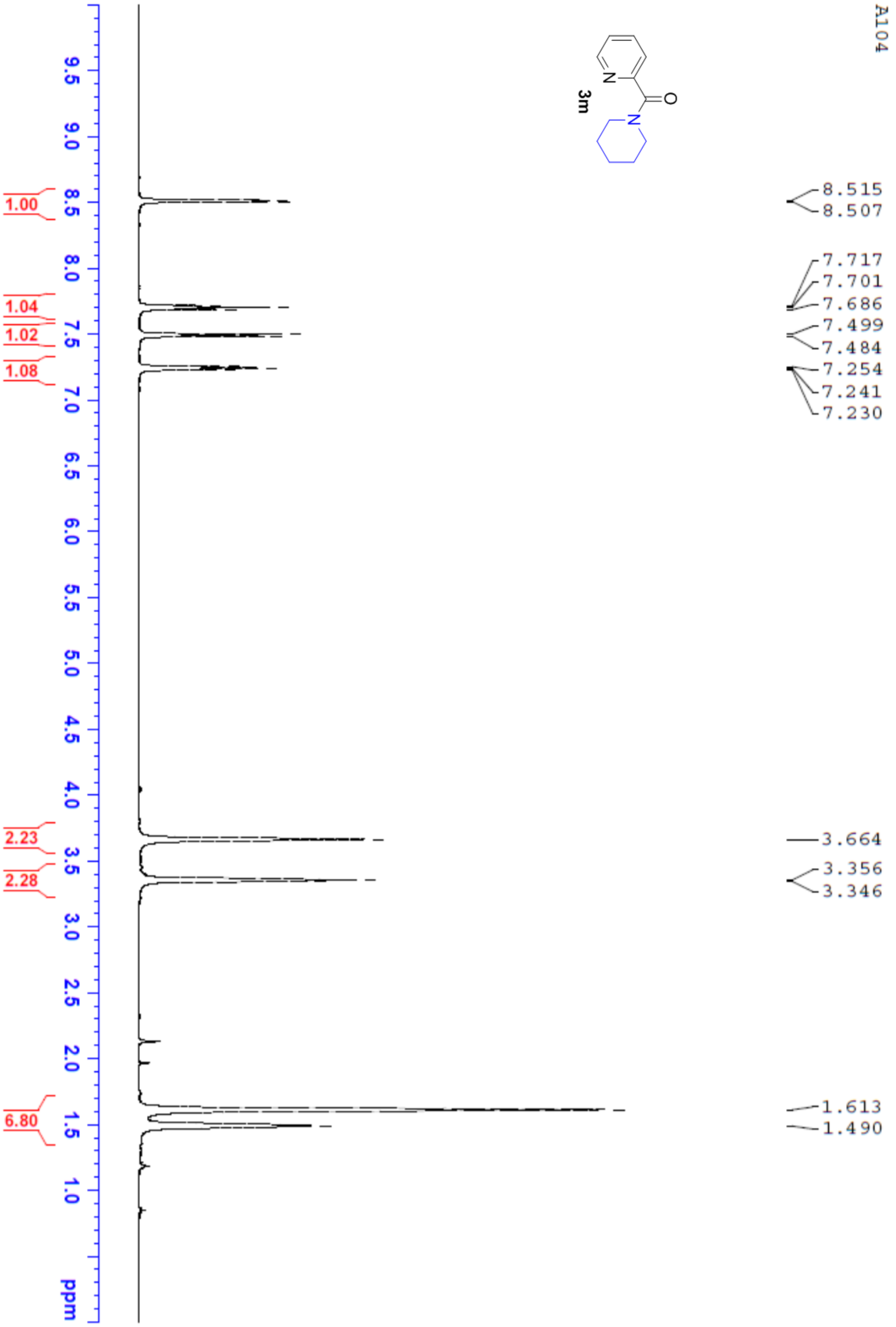
A104
3m
8.515
8.507
7.717
7.701
7.686
7.499
7.484
7.254
7.241
7.230
3.664
3.356
3.346
1.613
1.490
1.00
1.04
1.02
1.08
2.23
2.28
6.80
9.5
9.0
8.5
8.0
7.5
7.0
6.5
6.0
5.5
5.0
4.5
4.0
3.5
3.0
2.5
2.0
1.5
1.0
ppm

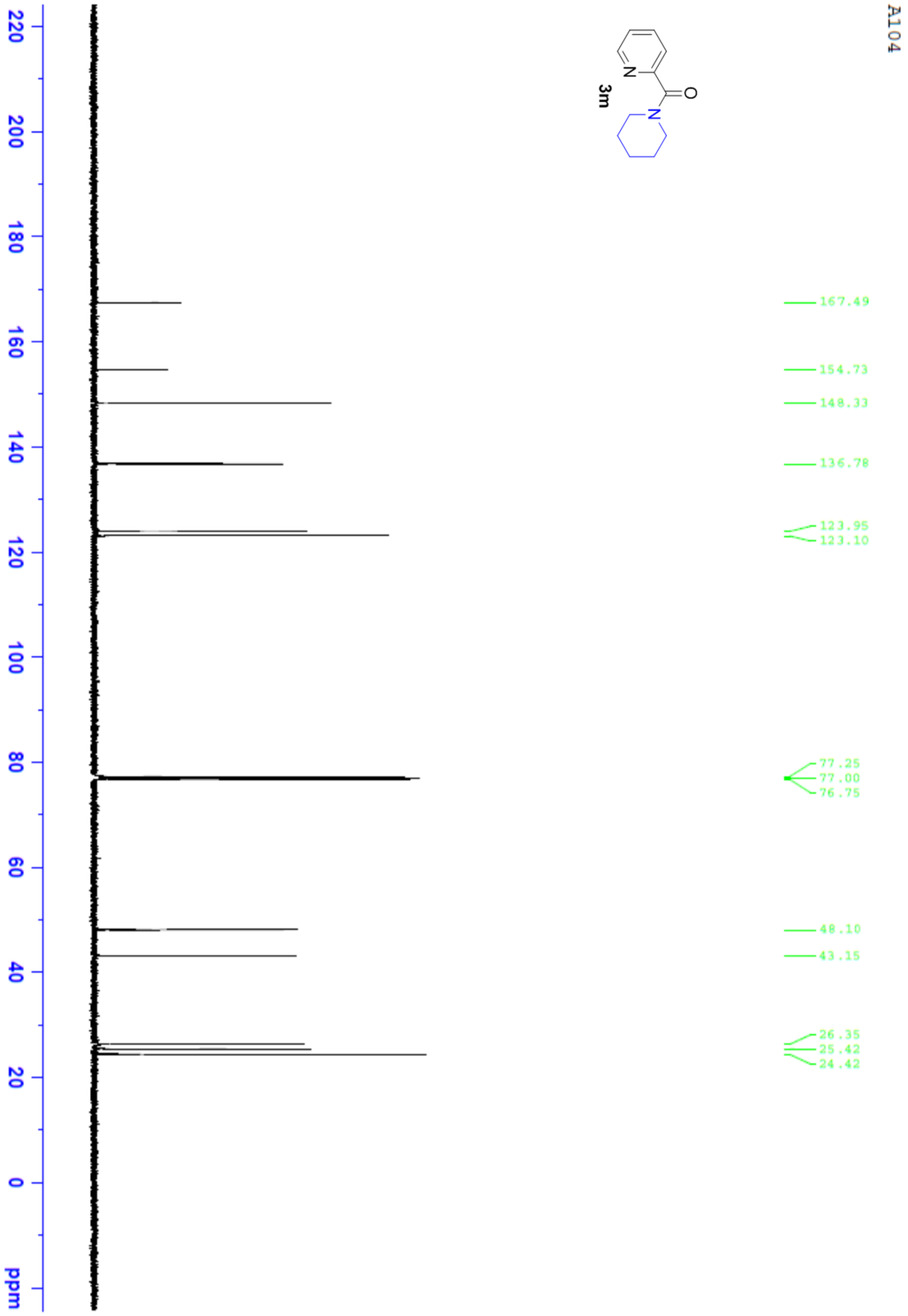
A104
3m
167.49
154.73
148.33
136.78
123.95
123.10
77.25
77.00
76.75
48.10
43.15
26.35
25.42
24.42
220
200
180
160
140
120
100
80
60
40
20
0
ppm

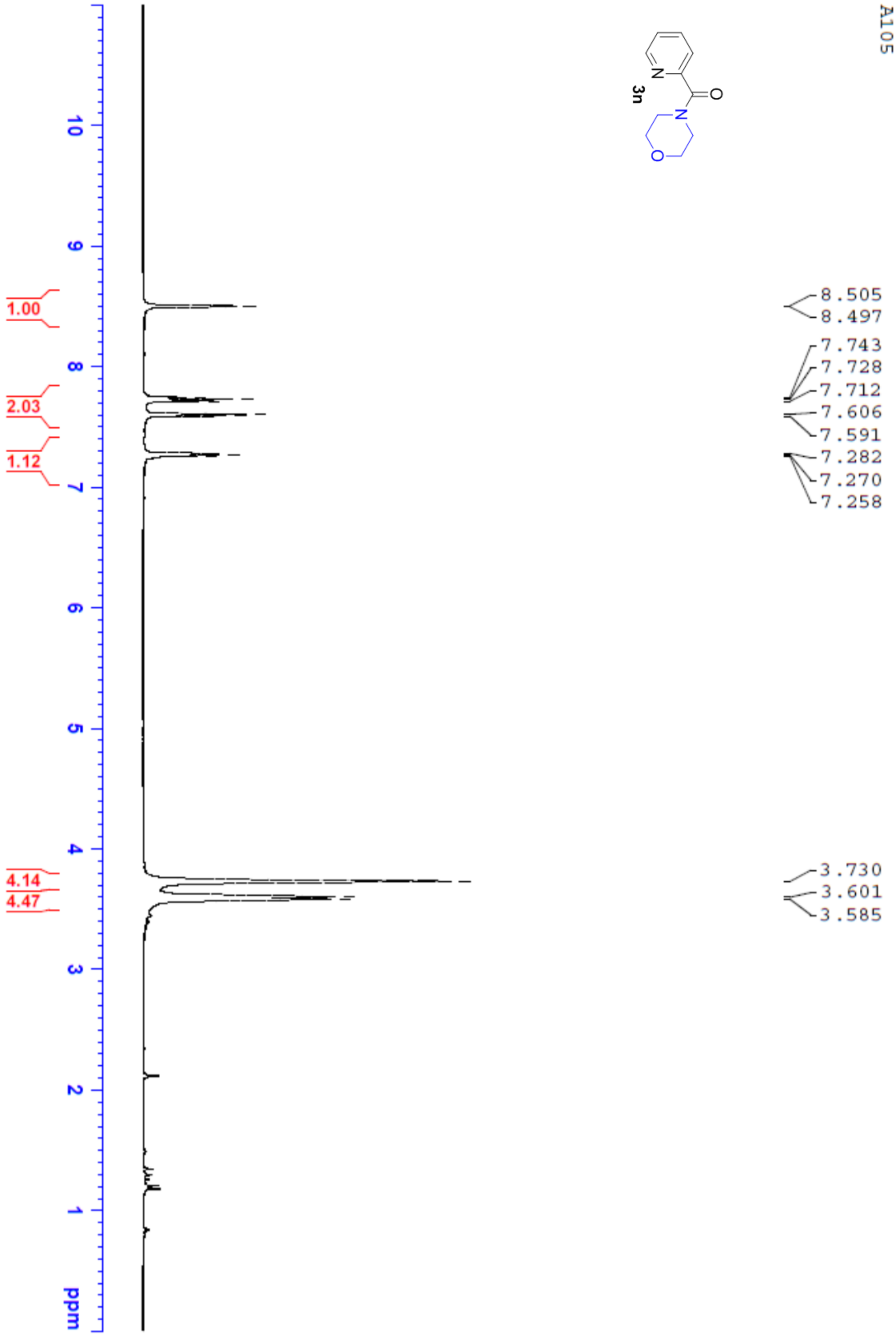
A105
3n
8.505
8.497
7.743
7.728
7.712
7.606
7.591
7.282
7.270
7.258
3.730
3.601
3.585
1.00
2.03
1.12
4.14
4.47
ppm

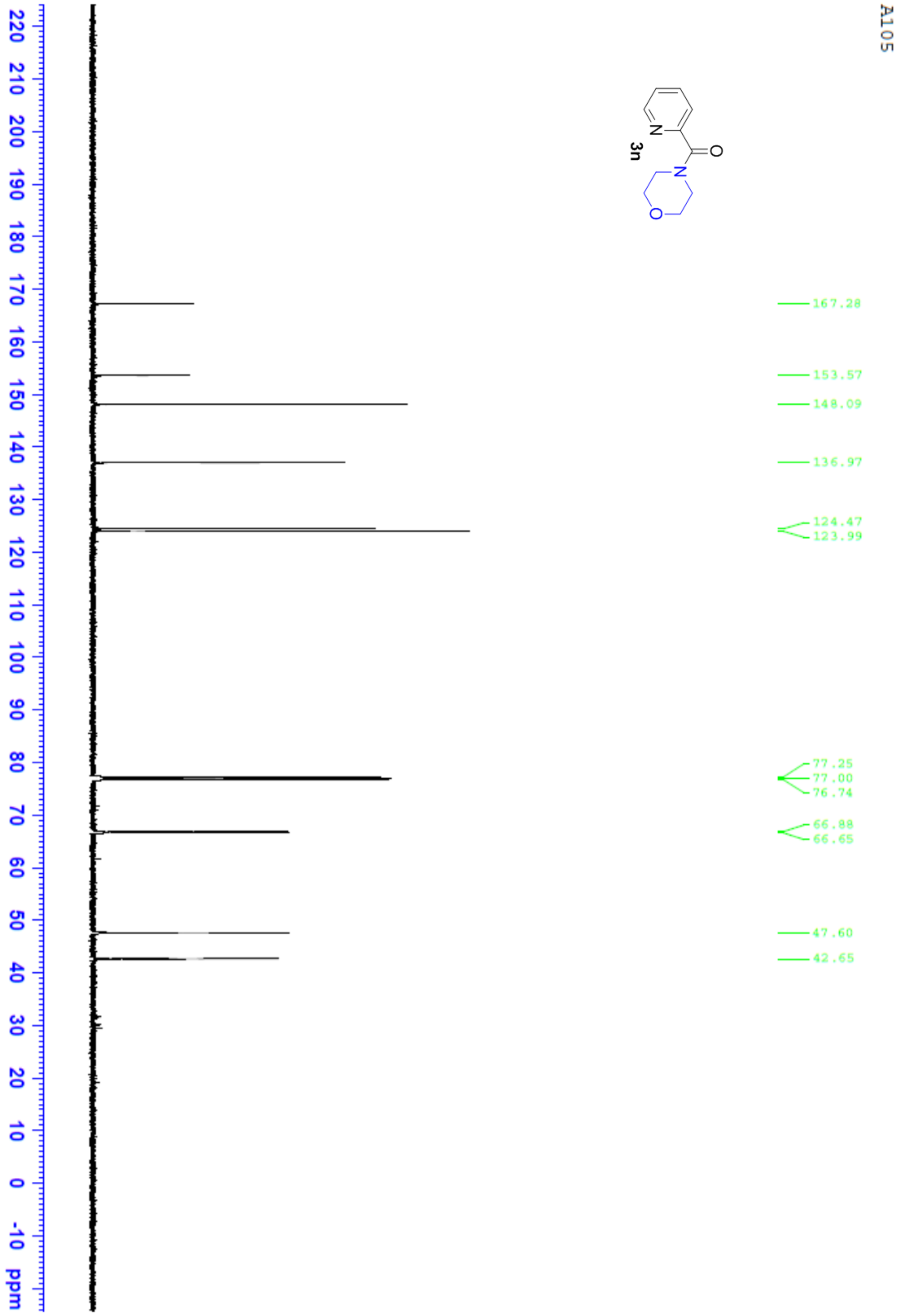
A105
3n
167.28
153.57
148.09
136.97
124.47
123.99
77.25
77.00
76.74
66.88
66.65
47.60
42.65
220 210 200 190 180 170 160 150 140 130 120 110 100 90 80 70 60 50 40 30 20 10 0 -10 ppm

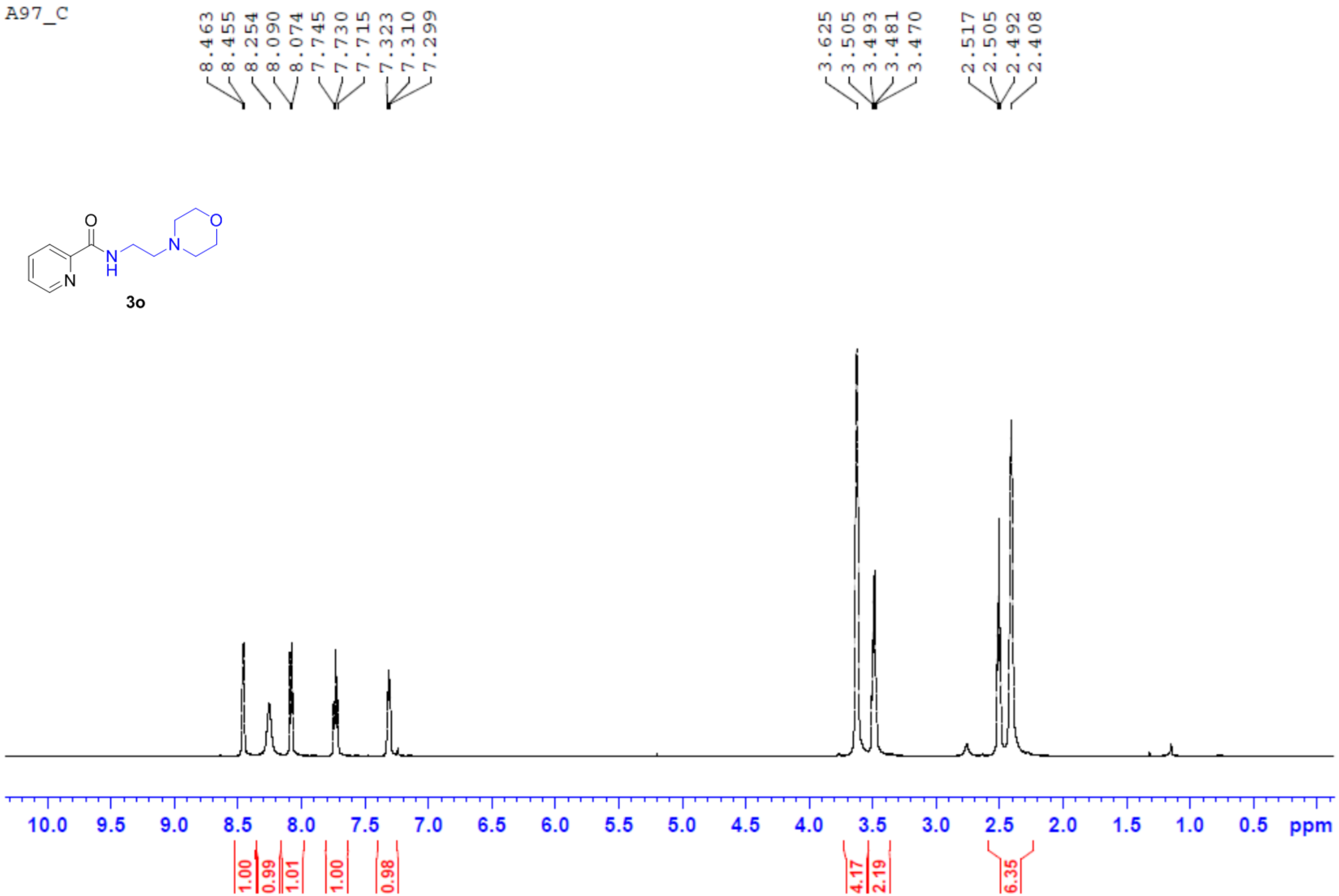
A97_C
8.463
8.455
8.254
8.090
8.074
7.745
7.730
7.715
7.323
7.310
7.299
3.625
3.505
3.493
3.481
3.470
2.517
2.505
2.492
2.408
O
N
H
N
N
O
10.0 9.5 9.0 8.5 8.0 7.5 7.0 6.5 6.0 5.5 5.0 4.5 4.0 3.5 3.0 2.5 2.0 1.5 1.0 0.5 ppm
1.00
0.99
1.01
1.00
0.98
4.17
2.19
6.35

3o

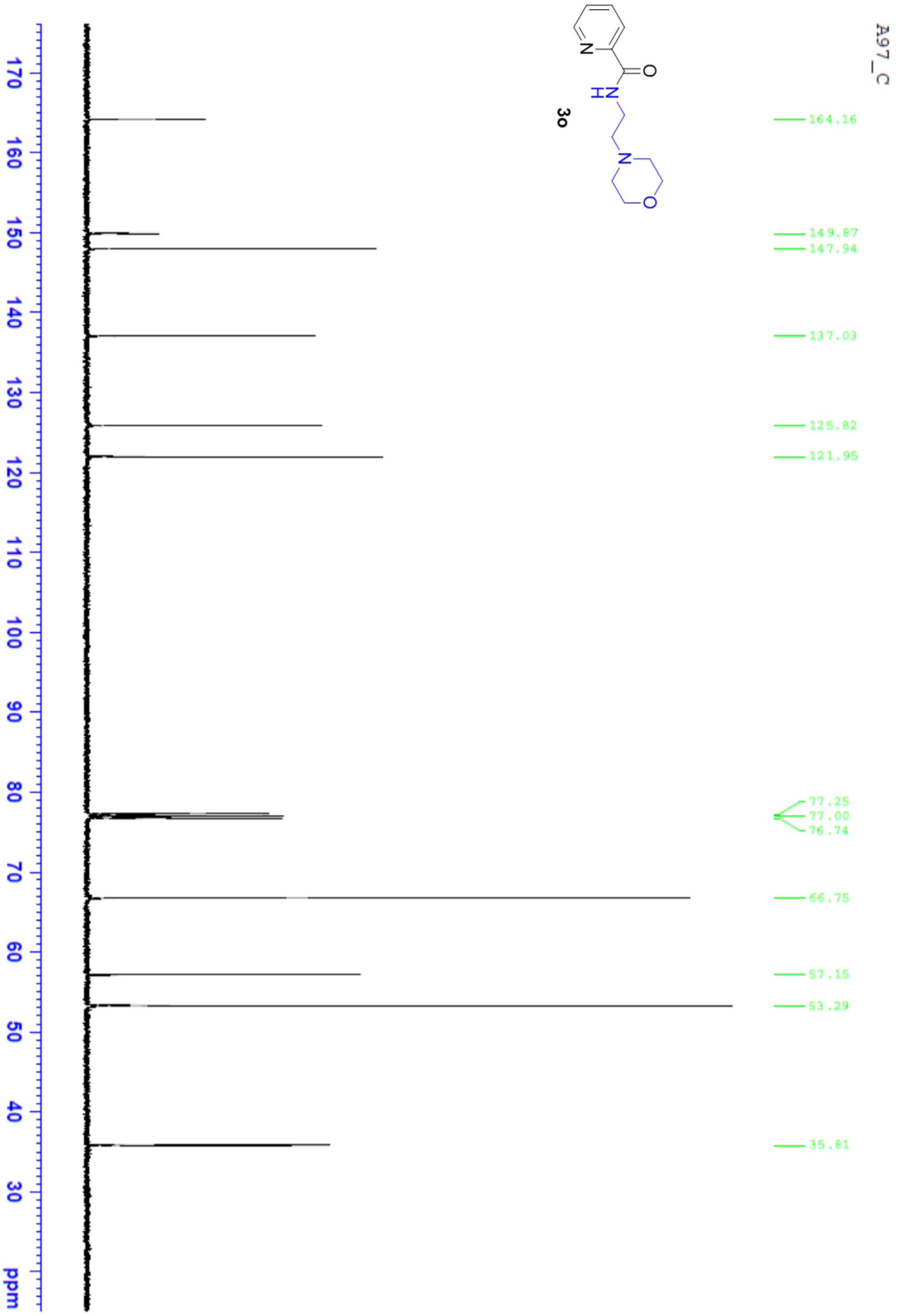
A97_C
3o
164.16
149.87
147.94
137.03
125.82
121.95
77.25
77.00
76.74
66.75
57.15
53.29
35.81
ppm

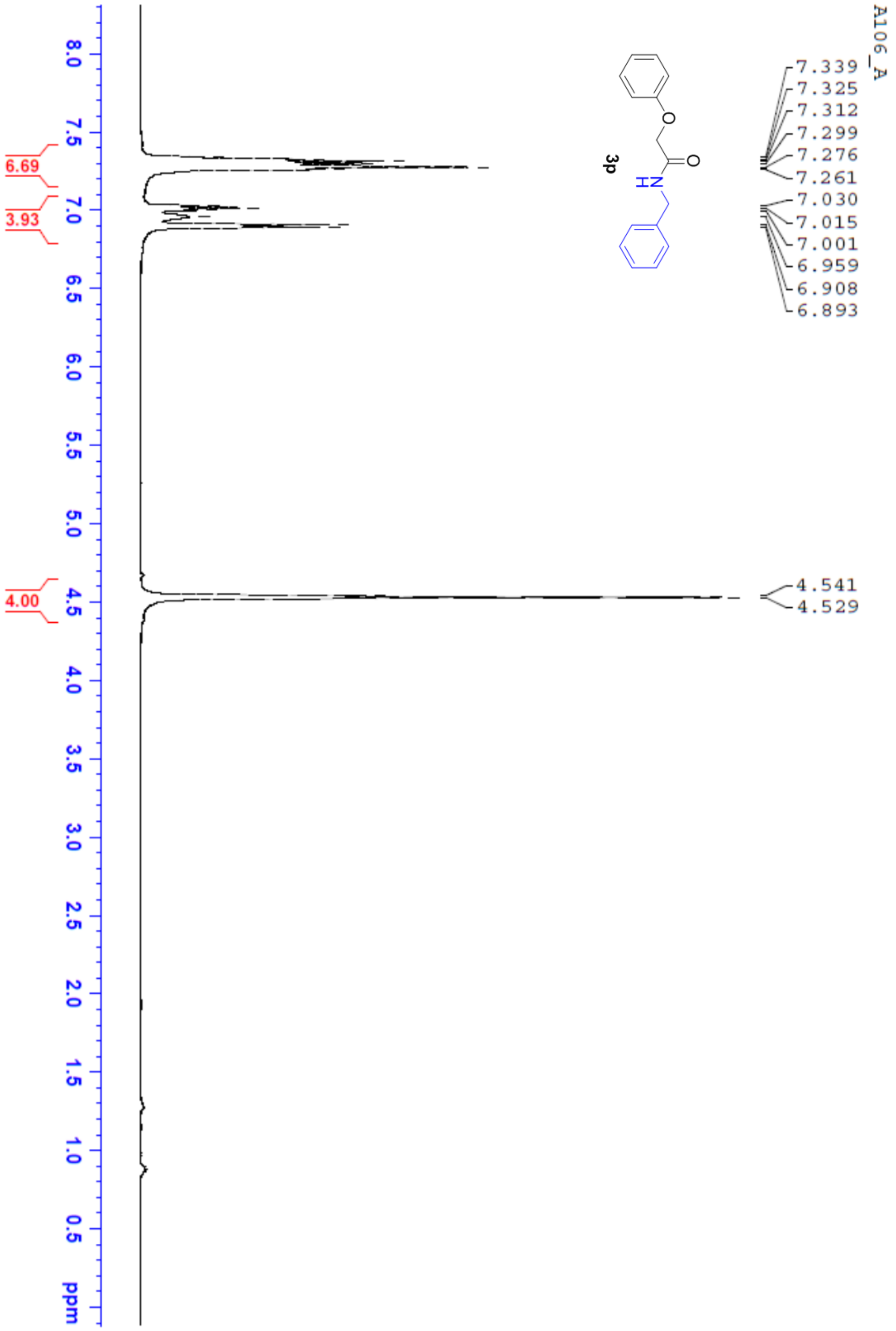

A106_A
7.339
7.325
7.312
7.299
7.276
7.261
7.030
7.015
7.001
6.959
6.908
6.893
4.541
4.529
3p
6.69
3.93
4.00
8.0
7.5
7.0
6.5
6.0
5.5
5.0
4.5
4.0
3.5
3.0
2.5
2.0
1.5
1.0
0.5
ppm

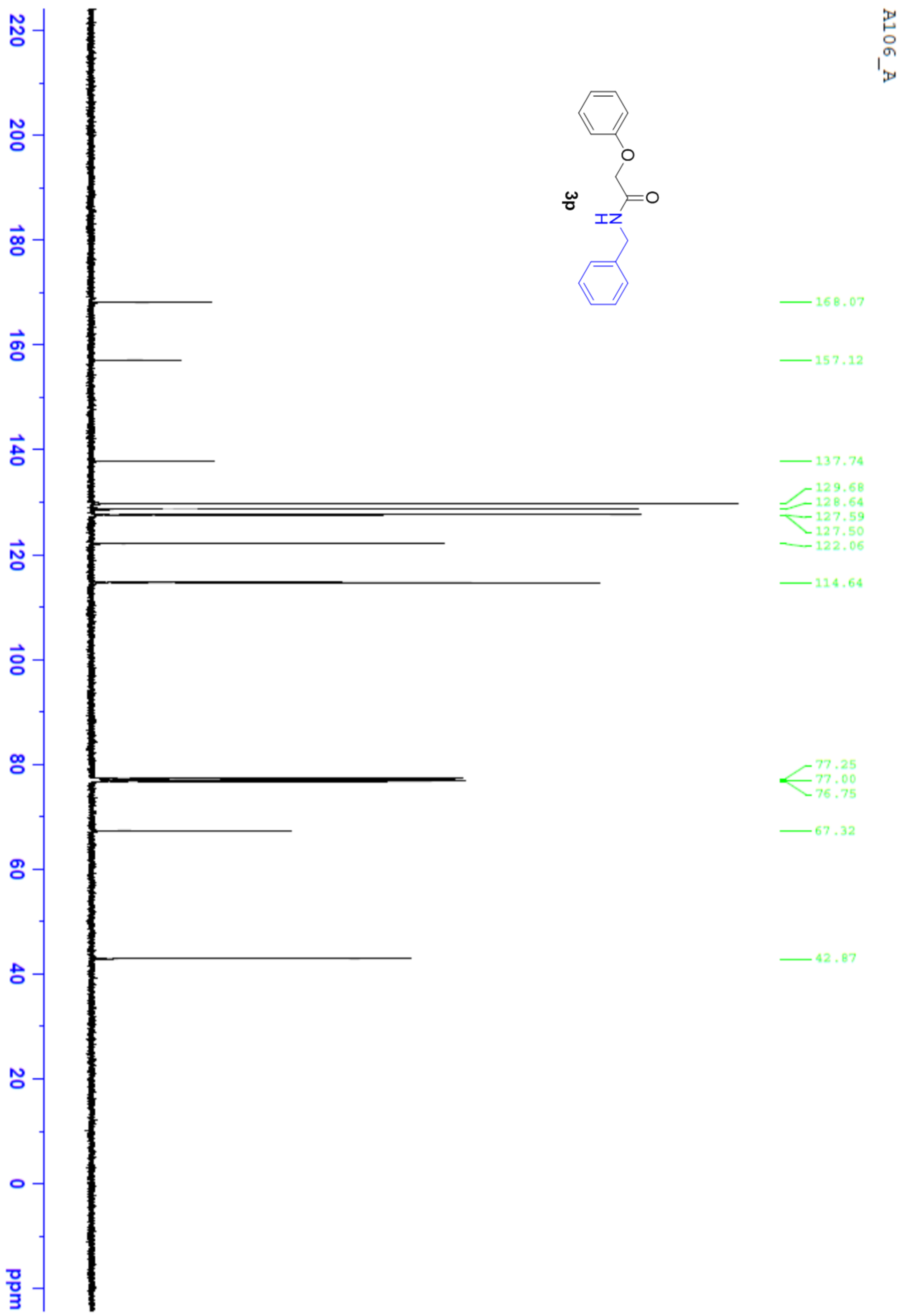
A106_A
3p
168.07
157.12
137.74
129.68
128.64
127.59
127.50
122.06
114.64
77.25
77.00
76.75
67.32
42.87
220
200
180
160
140
120
100
80
60
40
20
0
ppm

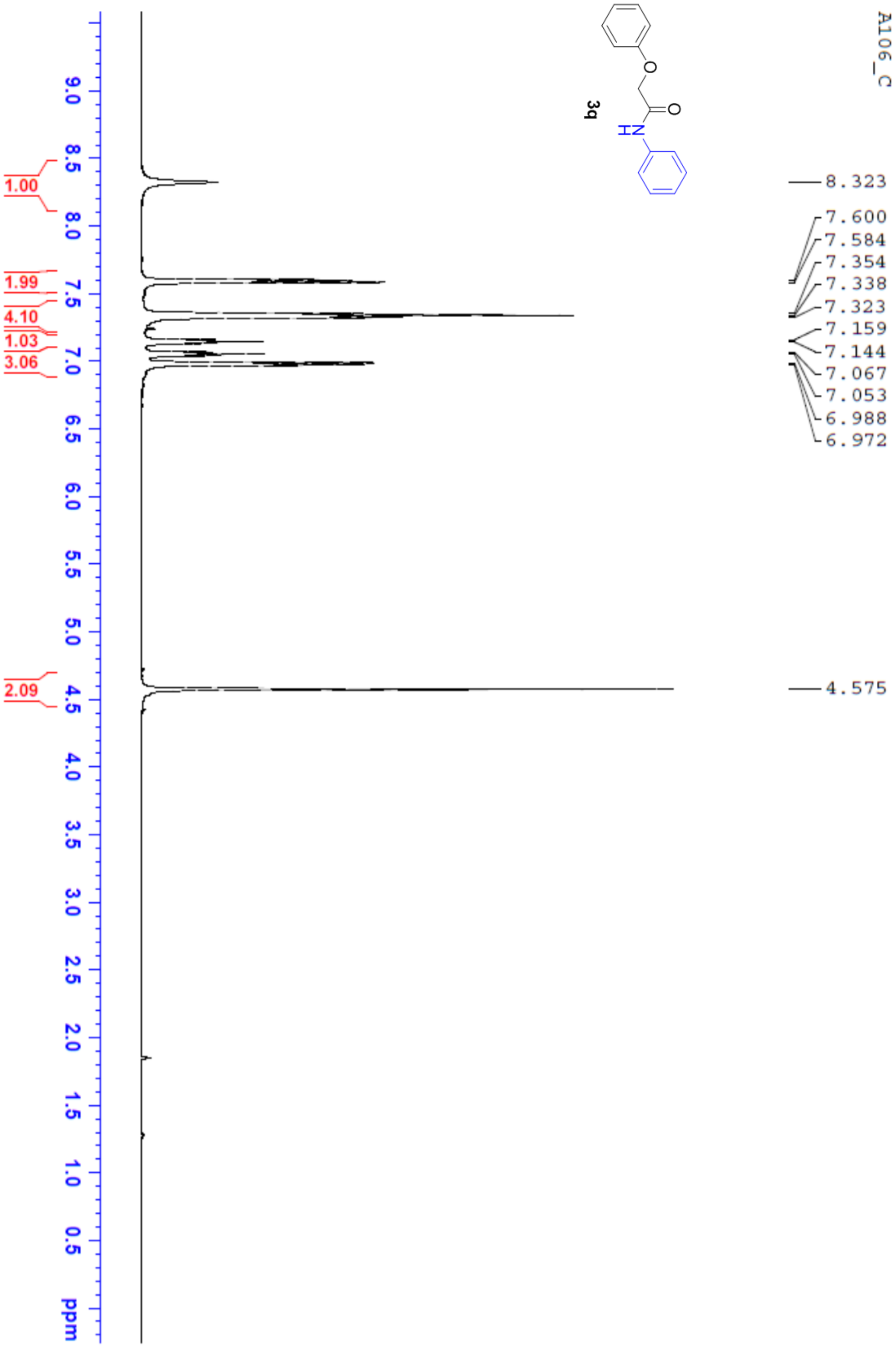
A106_C
3q
8.323
7.600
7.584
7.354
7.338
7.323
7.159
7.144
7.067
7.053
6.988
6.972
4.575
1.00
1.99
4.10
1.03
3.06
2.09
9.0
8.5
8.0
7.5
7.0
6.5
6.0
5.5
5.0
4.5
4.0
3.5
3.0
2.5
2.0
1.5
1.0
0.5
ppm

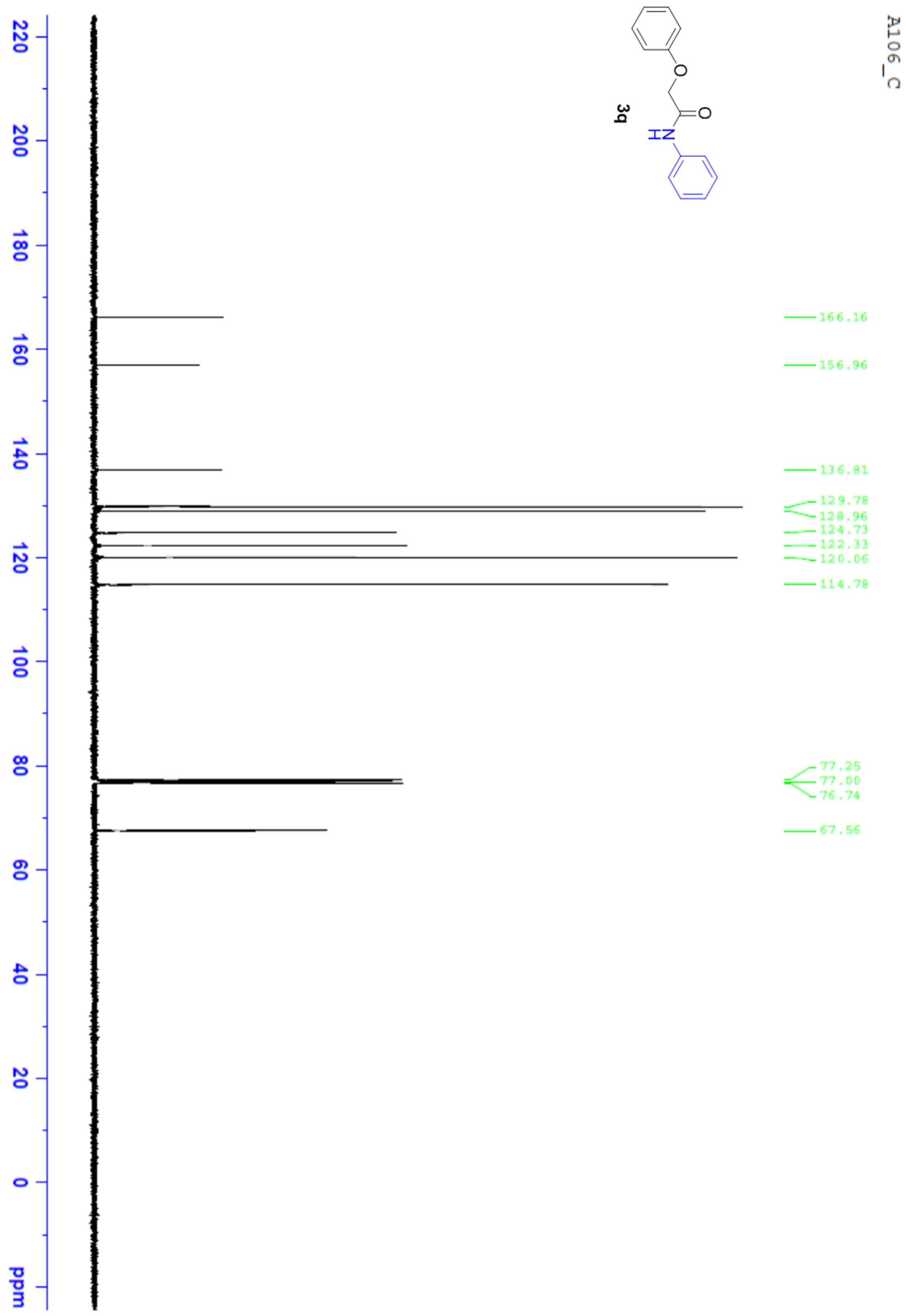
A106_C
3q
166.16
156.96
136.81
129.78
128.96
124.73
122.33
120.06
114.78
77.25
77.00
76.74
67.56
220
200
180
160
140
120
100
80
60
40
20
0
ppm

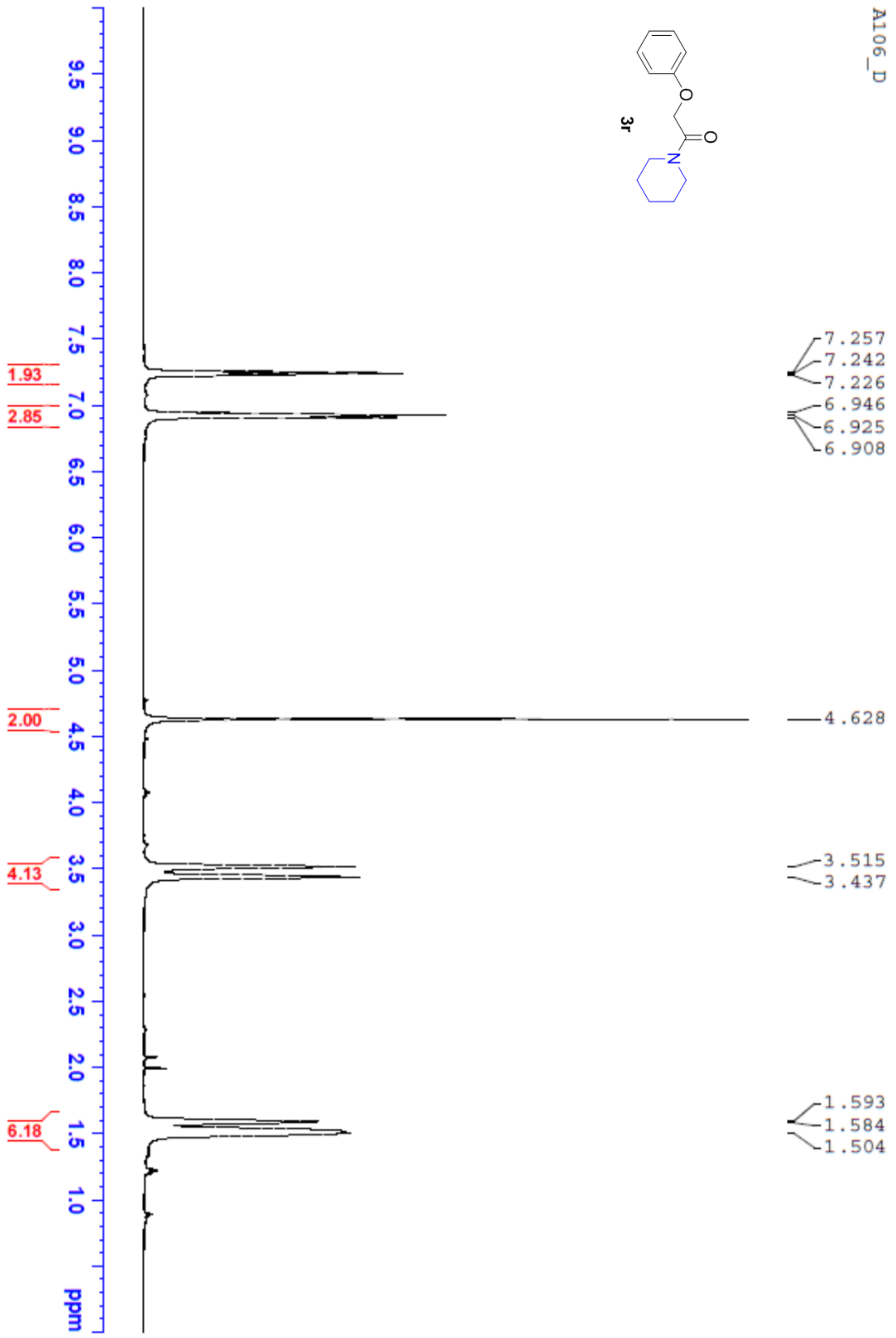
A106_D
3r
7.257
7.242
7.226
6.946
6.925
6.908
4.628
3.515
3.437
1.593
1.584
1.504
1.93
2.85
2.00
4.13
6.18
ppm

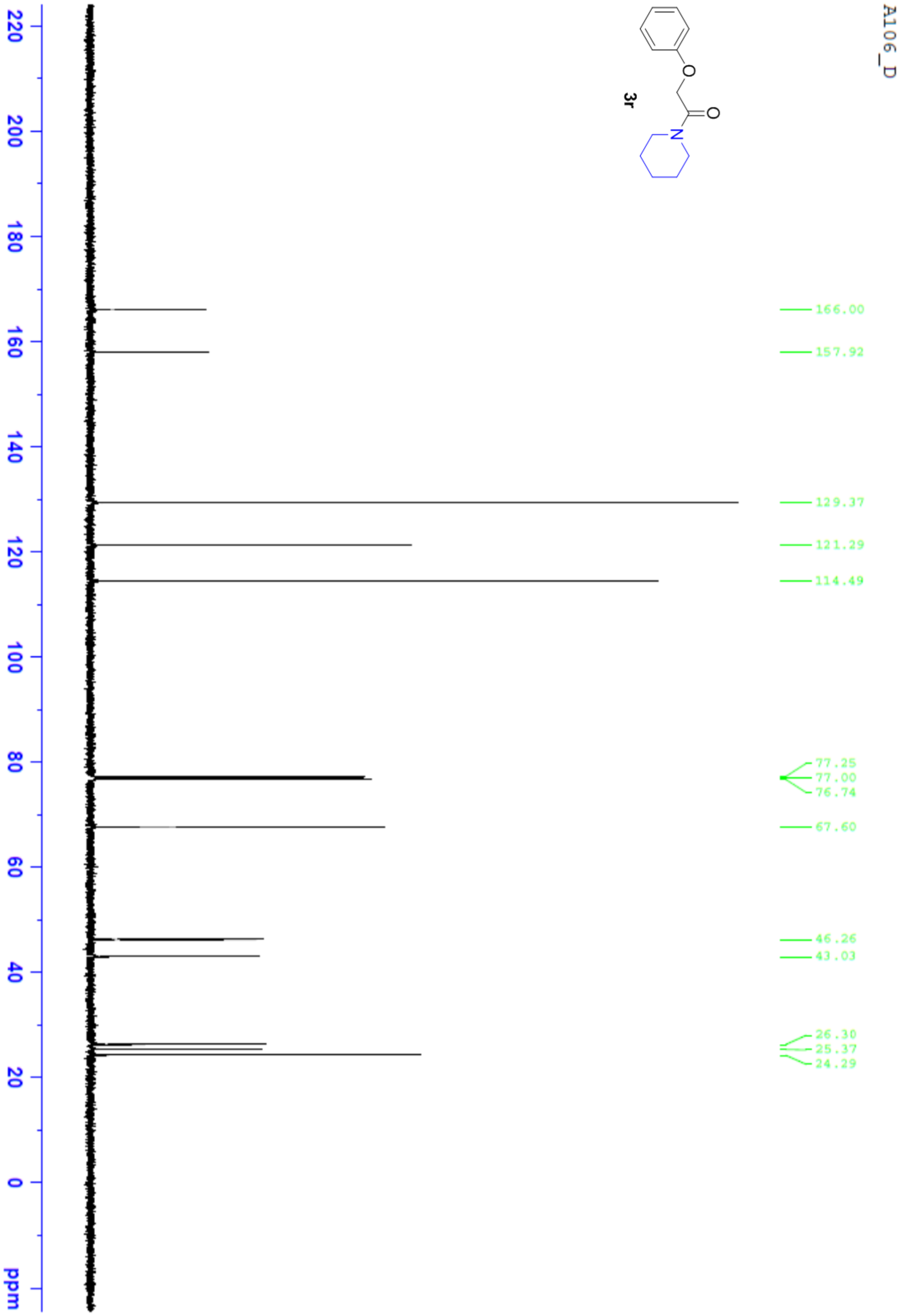
A106_D
3r
166.00
157.92
129.37
121.29
114.49
77.25
77.00
76.74
67.60
46.26
43.03
26.30
25.37
24.29
220
200
180
160
140
120
100
80
60
40
20
0
ppm

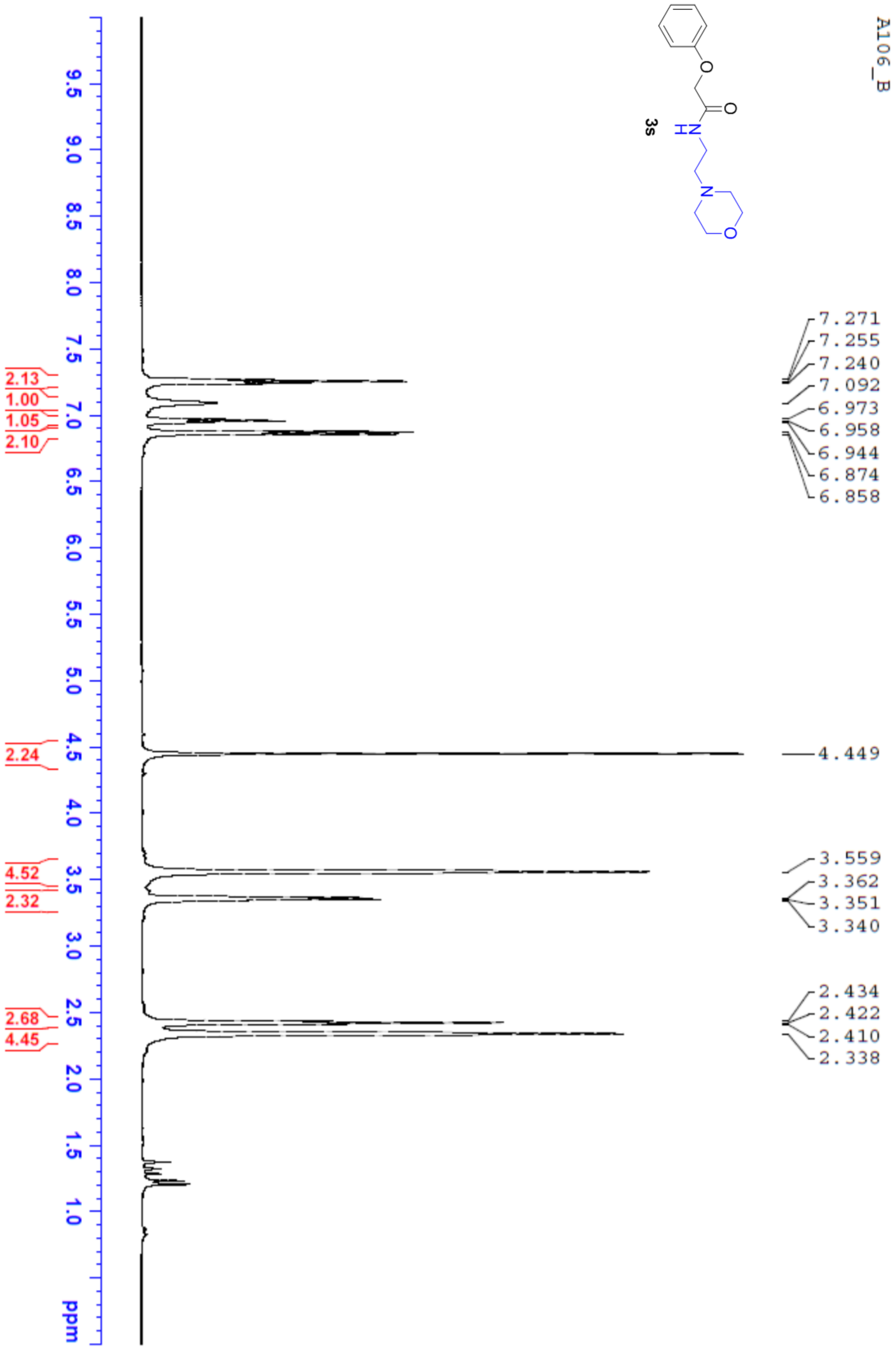
A106_B
3s
7.271
7.255
7.240
7.092
6.973
6.958
6.944
6.874
6.858
4.449
3.559
3.362
3.351
3.340
2.434
2.422
2.410
2.338
2.13
1.00
1.05
2.10
2.24
4.52
2.32
2.68
4.45
9.5
9.0
8.5
8.0
7.5
7.0
6.5
6.0
5.5
5.0
4.5
4.0
3.5
3.0
2.5
2.0
1.5
1.0
ppm

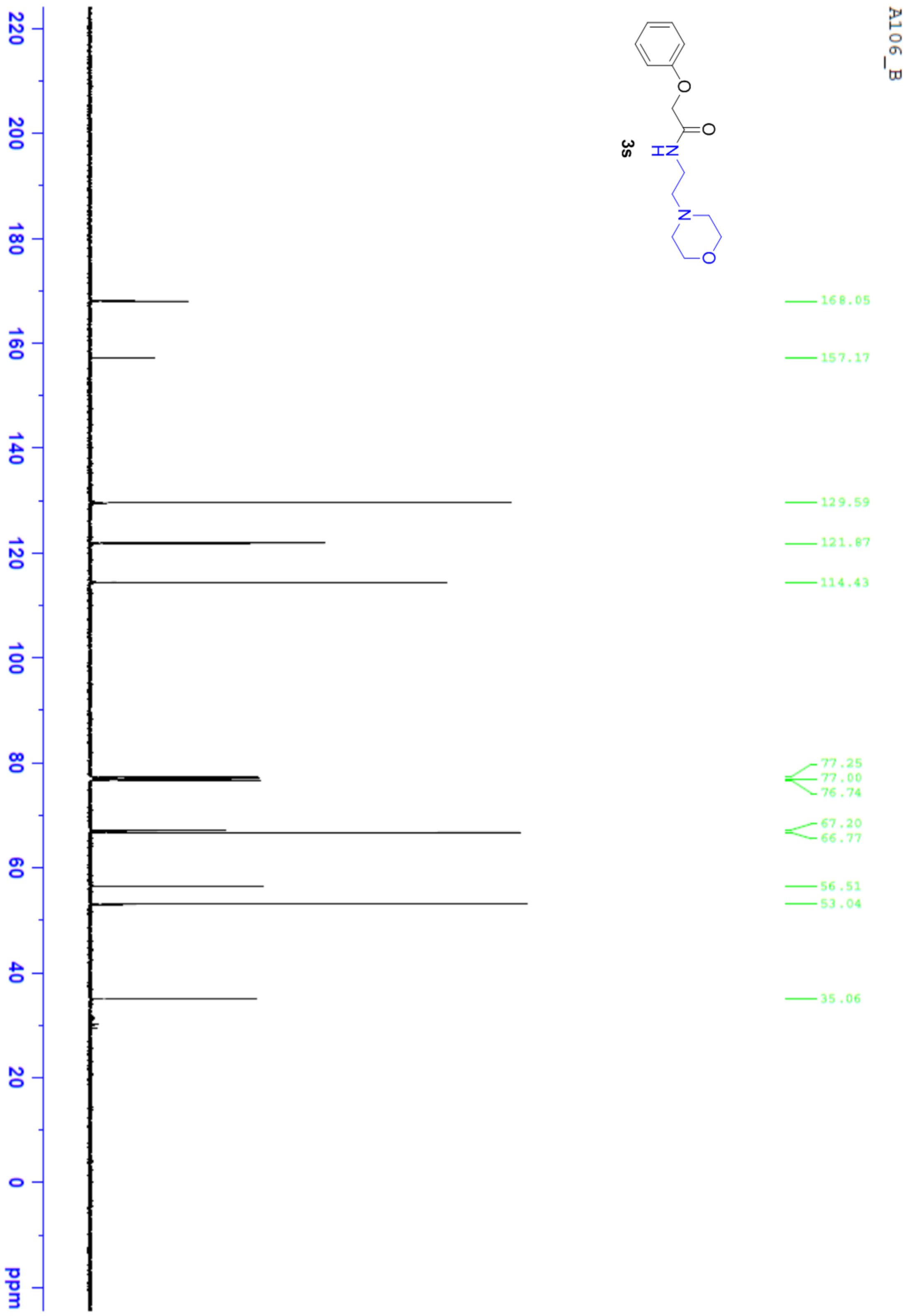
A106_B
3s
168.05
157.17
129.59
121.87
114.43
77.25
77.00
76.74
67.20
66.77
56.51
53.04
35.06
ppm

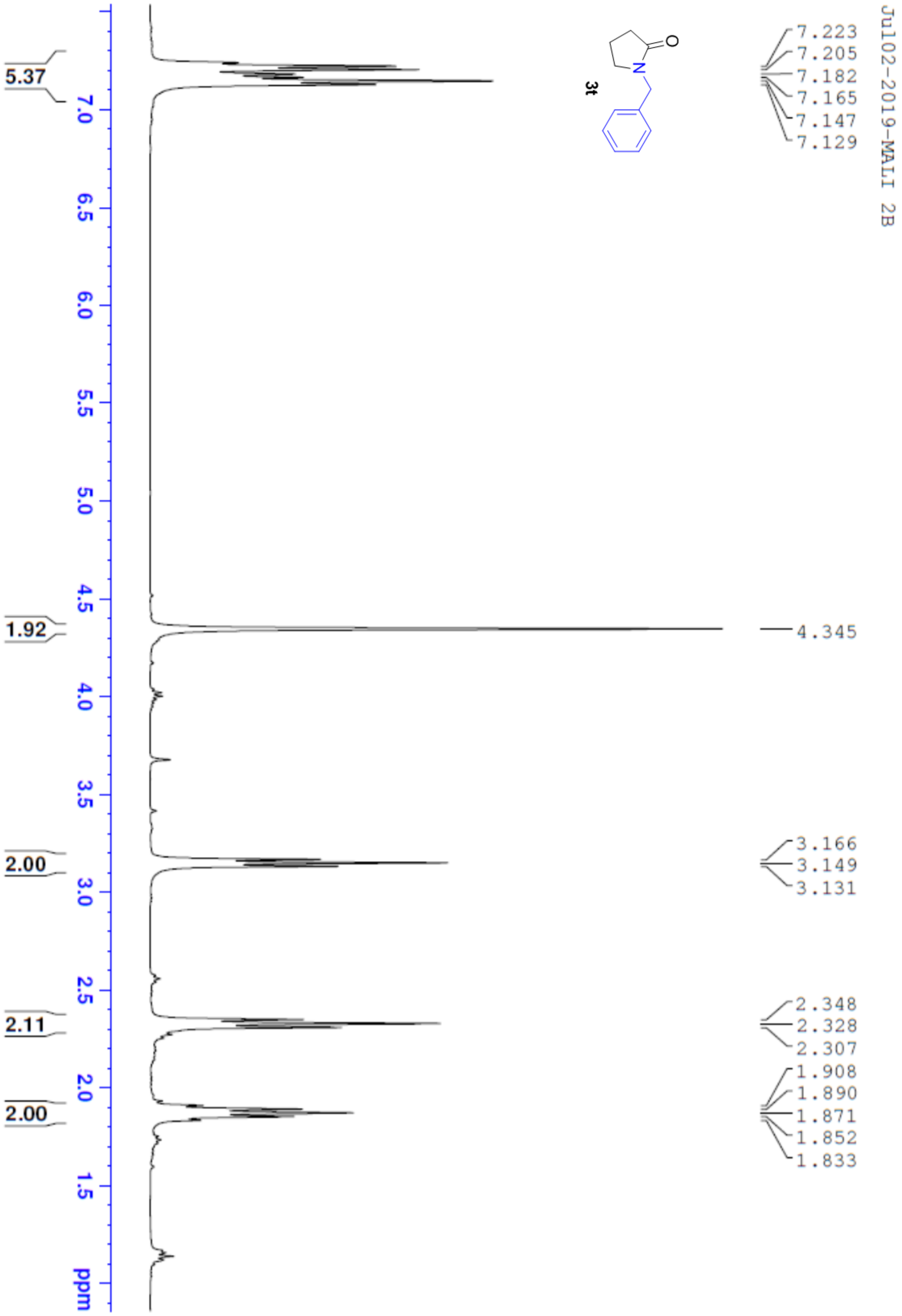
Jul02-2019-MALI 2B
7.223
7.205
7.182
7.165
7.147
7.129
4.345
3.166
3.149
3.131
2.348
2.328
2.307
1.908
1.890
1.871
1.852
1.833
3t
5.37
1.92
2.00
2.11
2.00
ppm

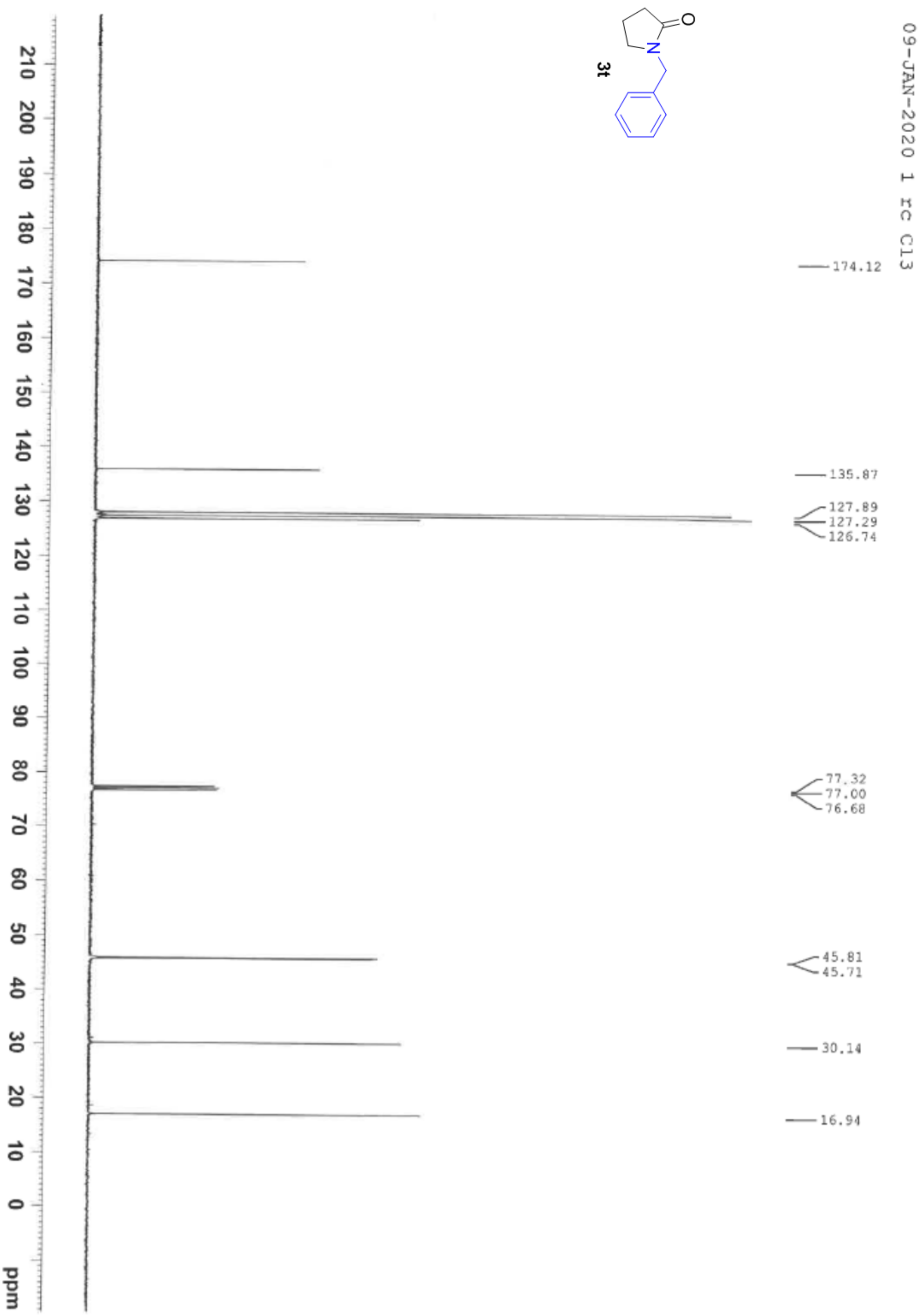

09-JAN-2020 1 rc C13
3t
174.12
135.87
127.89
127.29
126.74
77.32
77.00
76.68
45.81
45.71
30.14
16.94
210 200 190 180 170 160 150 140 130 120 110 100 90 80 70 60 50 40 30 20 10 0
ppm

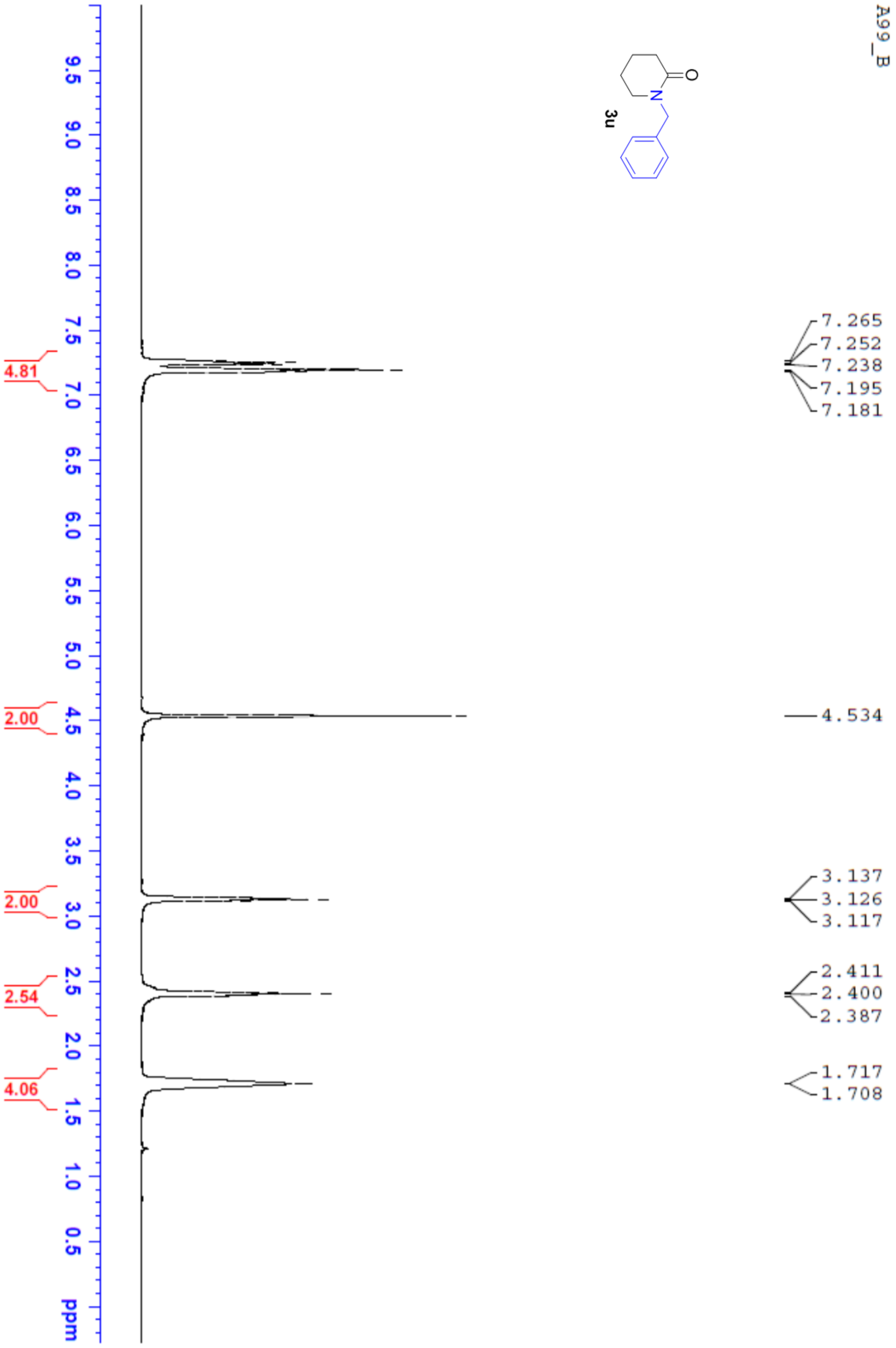
A99_B
3u
7.265
7.252
7.238
7.195
7.181
4.534
3.137
3.126
3.117
2.411
2.400
2.387
1.717
1.708
4.81
2.00
2.00
2.54
4.06
ppm

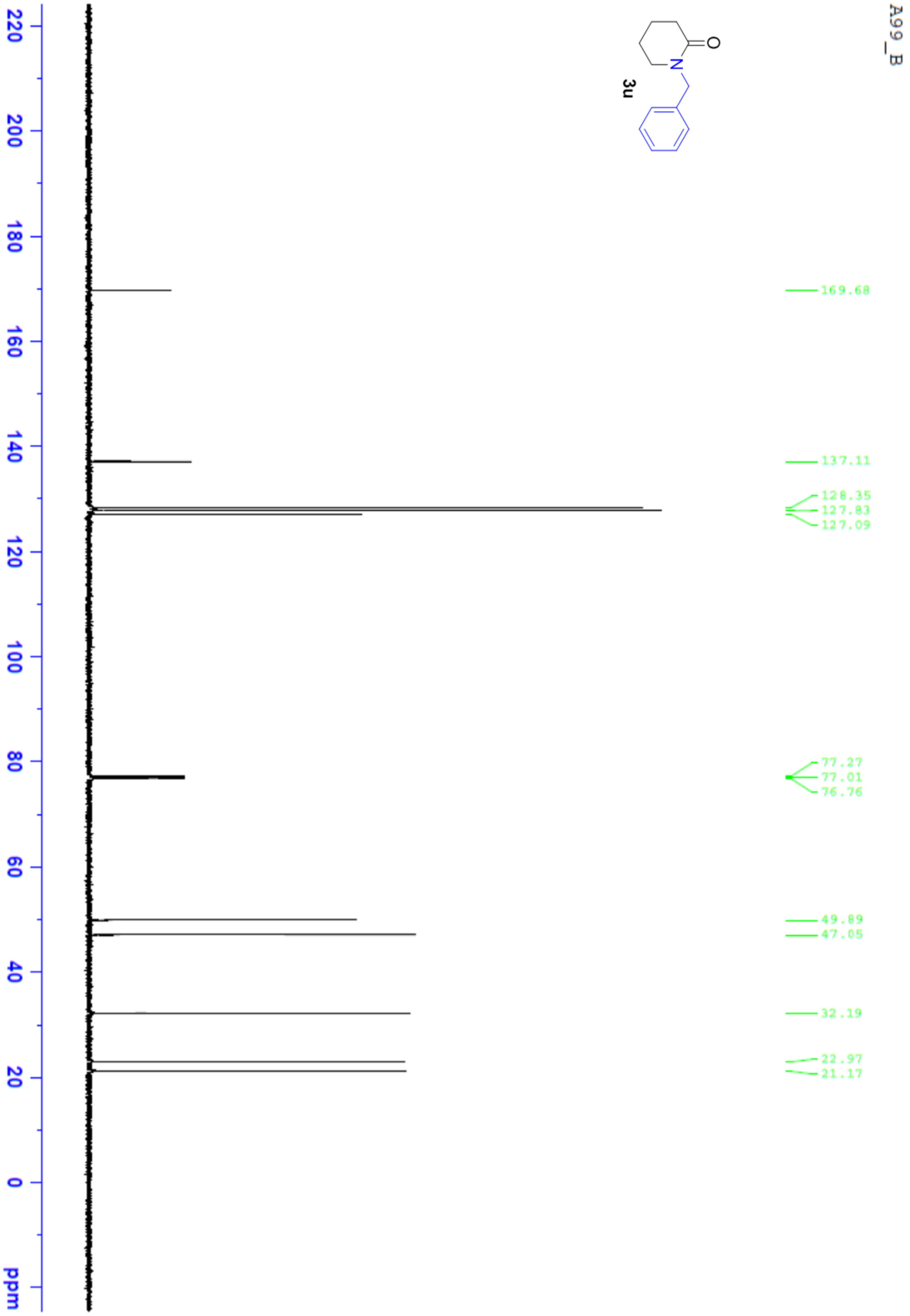
A99_B
3u
169.68
137.11
128.35
127.83
127.09
77.27
77.01
76.76
49.89
47.05
32.19
22.97
21.17
220
200
180
160
140
120
100
80
60
40
20
0
ppm

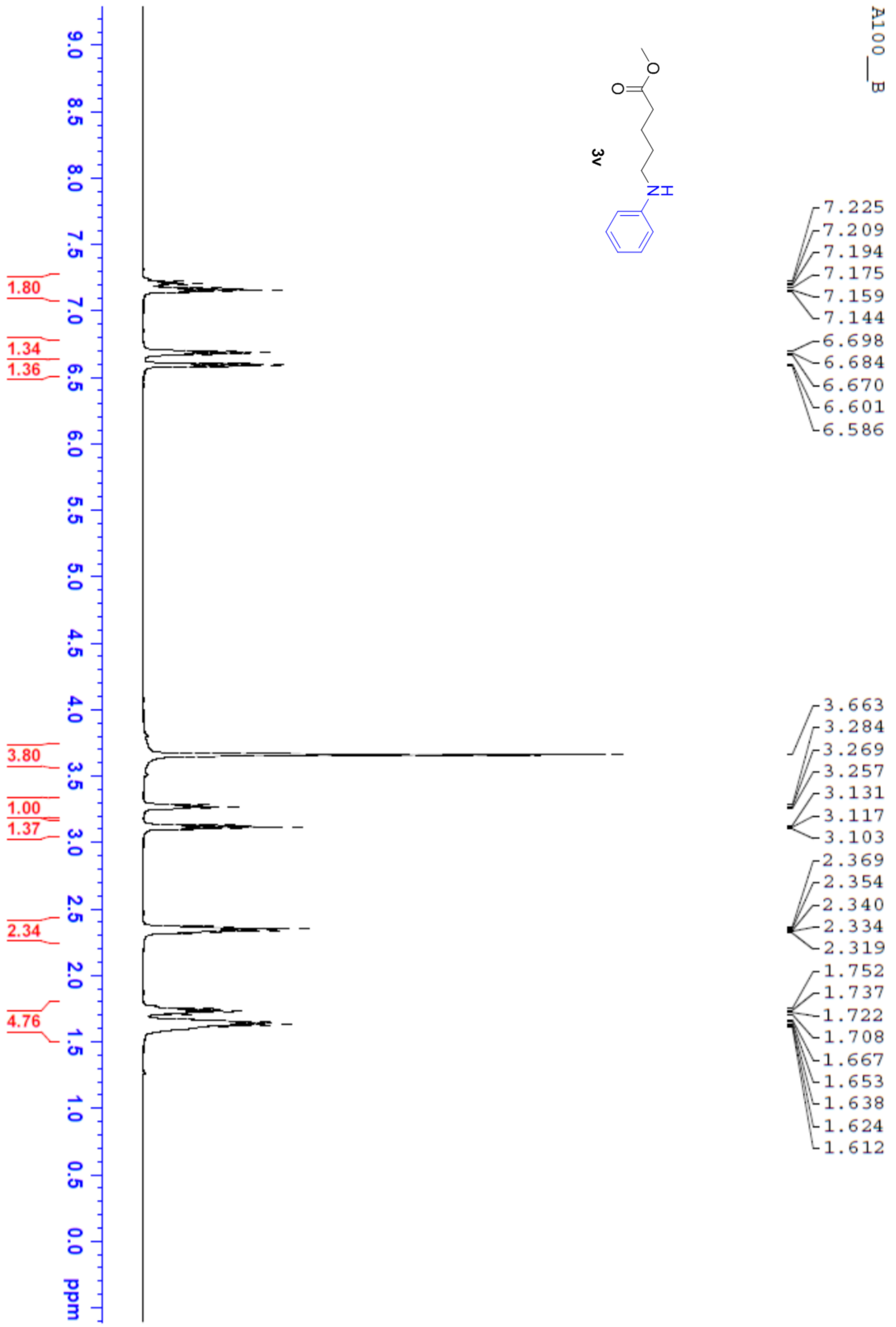

A100__B
3v
7.225
7.209
7.194
7.175
7.159
7.144
6.698
6.684
6.670
6.601
6.586
3.663
3.284
3.269
3.257
3.131
3.117
3.103
2.369
2.354
2.340
2.334
2.319
1.752
1.737
1.722
1.708
1.667
1.653
1.638
1.624
1.612
1.80
1.34
1.36
3.80
1.00
1.37
2.34
4.76
9.0
8.5
8.0
7.5
7.0
6.5
6.0
5.5
5.0
4.5
4.0
3.5
3.0
2.5
2.0
1.5
1.0
0.5
0.0
ppm

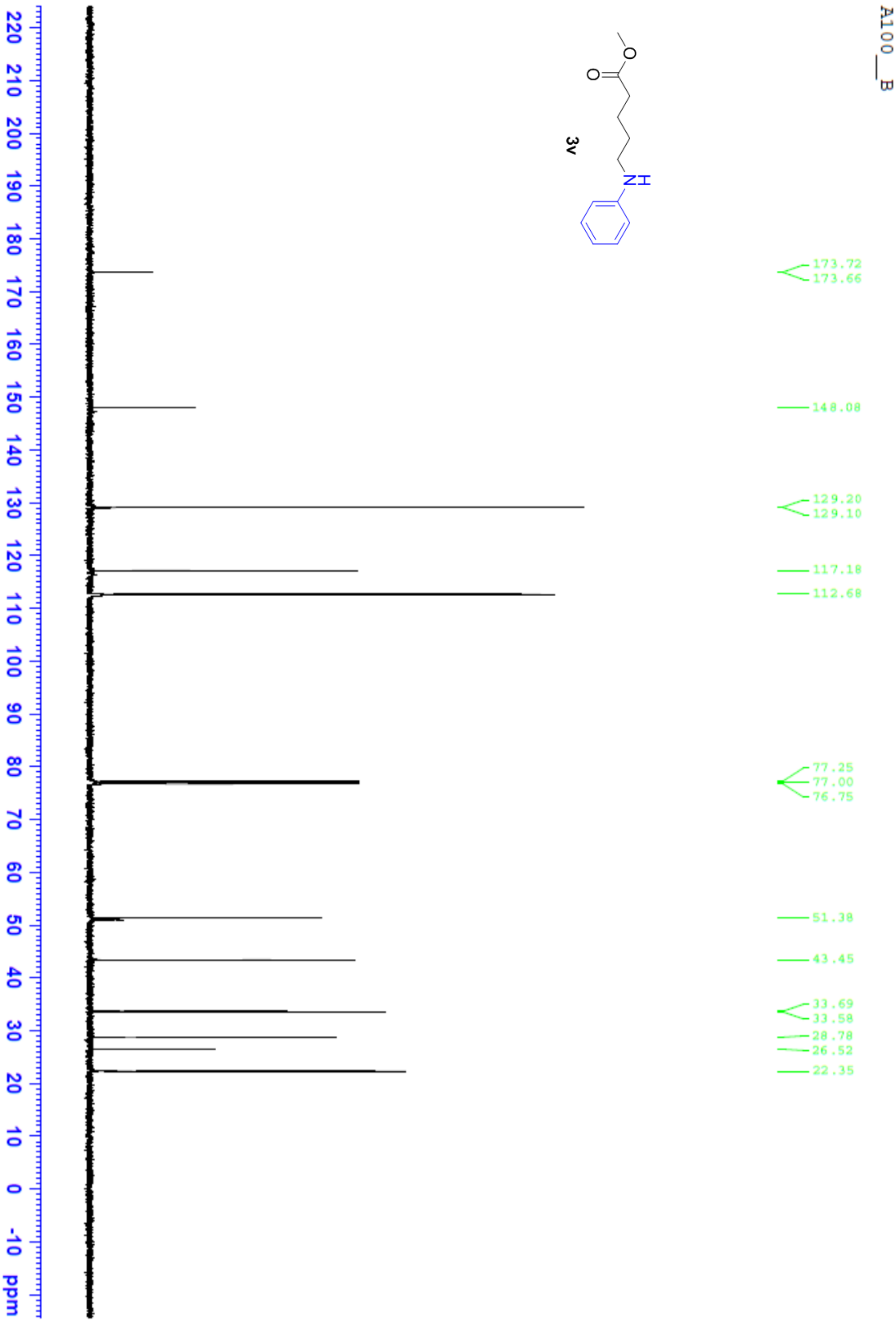
A100__B
3v
173.72
173.66
148.08
129.20
129.10
117.18
112.68
77.25
77.00
76.75
51.38
43.45
33.69
33.58
28.78
26.52
22.35
220 210 200 190 180 170 160 150 140 130 120 110 100 90 80 70 60 50 40 30 20 10 0 -10 ppm

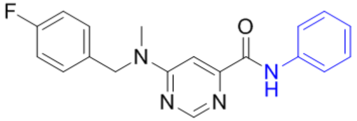

7

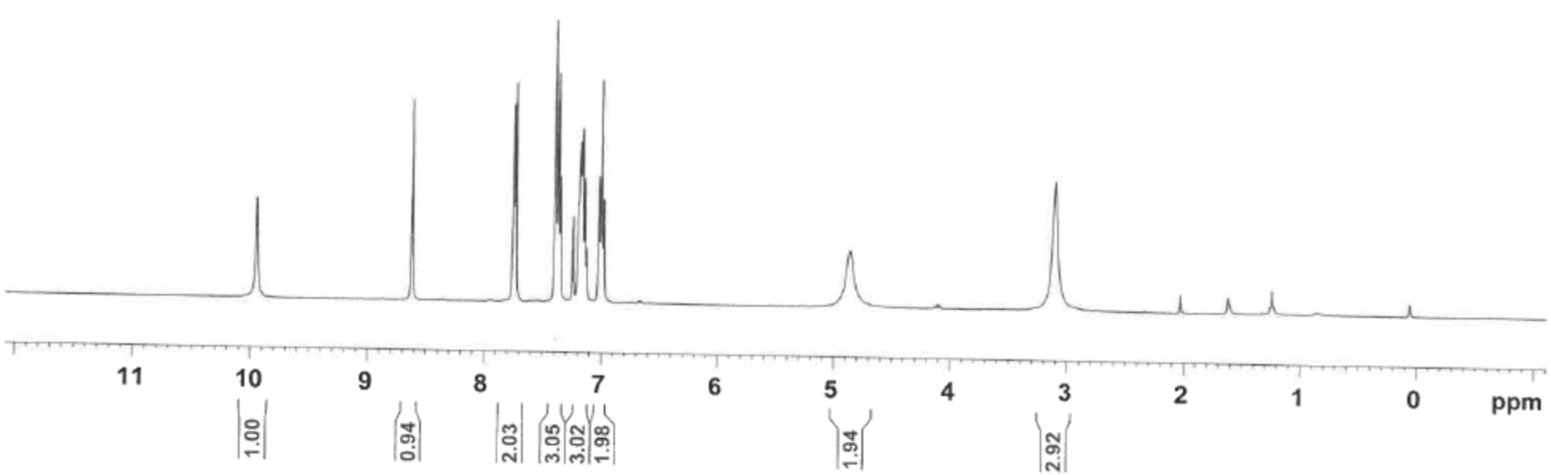
09-JAN-2020 rt 15 proton
9.945
8.617
7.749
7.729
7.394
7.368
7.348
7.240
7.195
7.178
7.162
7.141
7.122
7.020
6.999
6.977
4.862
3.105
1.00
0.94
2.03
3.05
3.02
1.98
1.94
2.92
ppm

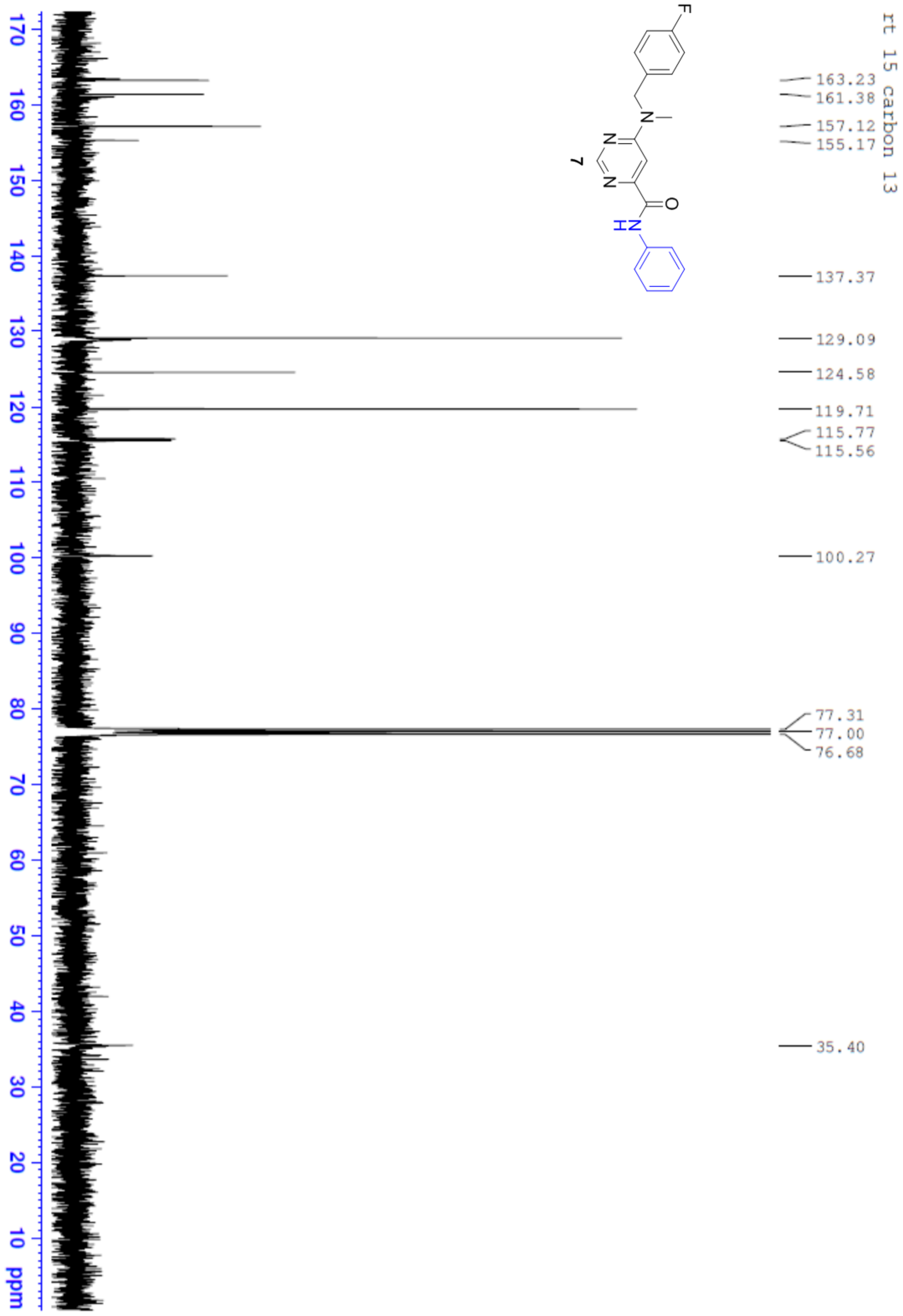
rt 15 carbon 13
163.23
161.38
157.12
155.17
137.37
129.09
124.58
119.71
115.77
115.56
100.27
77.31
77.00
76.68
35.40
7
ppm

rt 15 4F

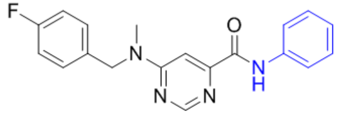

7

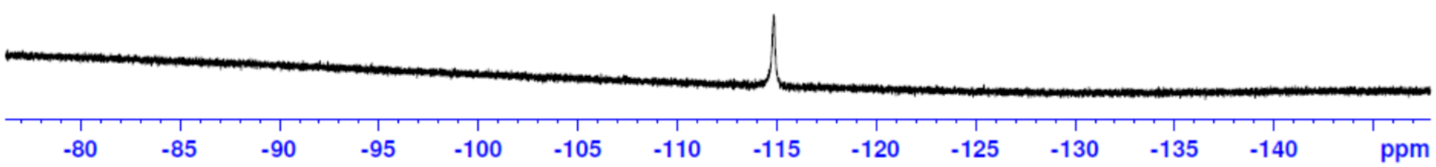


```
Current Data Parameters
NAME          rt 15  4F
EXPNO                 1
PROCNO                1

F2 - Acquisition Parameters
Date_          20200206
Time               9.12 h
INSTRUM           spect
PROBHD   Z108618_0217 (
PULPROG      zgfhigqn.2
TD               131072
SOLVENT           CDCl3
NS                  256
DS                    4
SWH           89285.711 Hz
FIDRES         1.362392 Hz
AQ            0.7340032 sec
RG                  575
DW                5.600 usec
DE                 6.50 usec
TE                299.0 K
D1           1.00000000 sec
D11          0.03000000 sec
D12          0.00002000 sec
TD0                   1
SFO1        376.4983502 MHz
NUC1                19F
P1                11.25 usec
PLW1        28.89800072 W
SFO2        400.1716007 MHz
NUC2                 1H
CPDPRG[2        waltz16
PCPD2             90.00 usec
PLW2        23.05500031 W
PLW12        0.40987000 W

F2 - Processing parameters
SI                65536
SF          376.5360040 MHz
WDW                  EM
SSB      0
LB                 0.30 Hz
GB       0
PC                 1.00
```
